# Effects of adaptive scaffolding on performance, cognitive load and engagement in game-based learning: a randomized controlled trial

**DOI:** 10.1186/s12909-024-05698-3

**Published:** 2024-08-29

**Authors:** Tjitske J. E. Faber, Mary E. W. Dankbaar, Walter W. van den Broek, Laura J. Bruinink, Marije Hogeveen, Jeroen J. G. van Merriënboer

**Affiliations:** 1 Department of Anesthesiology, Pain and Palliative Medicine, Radboud University Medical Center, Huispostnummer 717, P.O. Box 9101, Nijmegen, 6500 HB The Netherlands; 2 Erasmus MC, University Medical Center Rotterdam, Institute for Medical Education Research Rotterdam, P.O. Box 2040, 3000 CA Rotterdam, The Netherlands; 3 Department of Neonatology, Radboud University Medical Center, Radboud Institute for Health Sciences, P.O. Box 9101, 6500 HB Nijmegen, The Netherlands; 4 School of Health Professions Education, Faculty of Health, Medicine and Life Sciences, Maastricht University, P.O. Box 616, 6200 MD Maastricht, The Netherlands

**Keywords:** Game-based learning, Serious games, Simulation, Adaptivity, Cognitive load theory, Instructional design

## Abstract

**Background:**

While game-based learning has demonstrated positive outcomes for some learners, its efficacy remains variable. Adaptive scaffolding may improve performance and self-regulation during training by optimizing cognitive load. Informed by cognitive load theory, this study investigates whether adaptive scaffolding based on interaction trace data influences learning performance, self-regulation, cognitive load, test performance, and engagement in a medical emergency game.

**Methods:**

Sixty-two medical students from three Dutch universities played six game scenarios. They received either adaptive or nonadaptive scaffolding in a randomized double-blinded matched pairs yoked control design. During gameplay, we measured learning performance (accuracy, speed, systematicity), self-regulation (self-monitoring, help-seeking), and cognitive load. Test performance was assessed in a live scenario assessment at 2- and 6–12-week intervals. Engagement was measured after completing all game scenarios.

**Results:**

Surprisingly, the results unveiled no discernible differences between the groups experiencing adaptive and nonadaptive scaffolding. This finding is attributed to the unexpected alignment between the nonadaptive scaffolding and the needs of the participants in 64.9% of the scenarios, resulting in coincidentally tailored scaffolding. Exploratory analyses suggest that, compared to nontailored scaffolding, tailored scaffolding improved speed, reduced self-regulation, and lowered cognitive load. No differences in test performance or engagement were found.

**Discussion:**

Our results suggest adaptive scaffolding may enhance learning by optimizing cognitive load. These findings underscore the potential of adaptive scaffolding within GBL environments, cultivating a more tailored and effective learning experience. To leverage this potential effectively, researchers, educators, and developers are recommended to collaborate from the outset of designing adaptive GBL or computer-based simulation experiences. This collaborative approach facilitates the establishment of reliable performance indicators and enables the design of suitable, preferably real-time, scaffolding interventions. Future research should confirm the effects of adaptive scaffolding on self-regulation and learning, taking care to avoid unintended tailored scaffolding in the research design.

**Trial registration:**

This study was preregistered with the Center for Open Science prior to data collection. The registry may be found at https://osf.io/7ztws/.

**Supplementary Information:**

The online version contains supplementary material available at 10.1186/s12909-024-05698-3.

## Introduction

Game-based learning (GBL) is a promising tool to support learning [1–3], but differences in effectiveness between learners and learner groups have been observed [4–6]. Adaptive scaffolding, meaning the automatic modulation of support measures based on players’ characteristics or behaviors, has been shown to improve learning outcomes [7, 8], possibly through the optimization of cognitive load [3, 9, 10]. However, the number of studies into the effects of adaptive scaffolding on cognitive load and learning outcomes in GBL is low [9–11]. This study aims to investigate the effects of adaptive scaffolding in a medical emergency simulation game.

## Theoretical background

### Cognitive load theory

To understand how the same instruction may have different effects on different learner groups, we turn to cognitive load theory (CLT [12]). This theory assumes a limited working memory and unlimited long-term memory holding cognitive schemas. Expertise comes from knowledge stored as schemas, and learning is described as the construction and automation of such schemas. To create schemas, new information must be ‘mindfully combined’ with other information or existing schemas. When working memory is overloaded, learning is impaired [13]. It follows that learners who have already developed relevant schemas will have more working memory resources to spare to deal with the task. These experienced learners may perform worse at a task when detailed instructions are provided (the “expertise reversal effect” [14]) because working memory becomes bogged down with attempts to cross-reference the instruction with existing schemas in long-term memory. Novice performers will benefit from instruction as the instruction may act as a central executive to organize the relevant information in working memory [3], freeing up cognitive load. Accordingly, instructional design should aim to 1) deliver learning activities, which present new information to be combined into more complex schemas (construction) or the opportunity to repeatedly apply existing schemas to new problems (automation), and 2) optimize cognitive load, to allow the learner to mindfully combine the new information.

In understanding how instruction influences cognitive load it is helpful to consider different types of cognitive load. *Intrinsic* cognitive load refers to the demands on working memory caused by the learning task itself. The more complex the learning task, or the lower the learner’s expertise, the higher the intrinsic cognitive load. Thus, the same learning task may cause a high cognitive load for a low-expertise learner but a low cognitive load for a high-expertise learner. *Extraneous* cognitive load is the load caused by demands on working memory caused by the instruction and the environment, rather than the information to be learned. Finally, *germane* cognitive load is the load required to deal with intrinsic cognitive load. It redistributes working memory resources to activities relevant to learning so that it promotes schema construction and automation. Techniques to measure cognitive load include direct measures such as subjective rating scales, including the popular 1-item Paas scale for mental effort [15–17], and dual-task methods (e.g. [18], Rojas, Haji [19], as well as indirect measures such as learning outcomes [20], physiological measures [21], and behavioral measures [22].

To optimize cognitive load in learning environments several principles have been described (e.g. [3, 23, 24]), including tailoring the instructional design to varying levels of learner expertise [9]. This may be accomplished through scaffolding, “the process whereby the support given to students is gradually reduced to counteract the adverse effects of excessive task complexity” [25]. Scaffolding is closely related to Vygotsky’s Zone of Proximal Development [26]. The additional support may take the form of *supportive* information (the provision of domain-general strategies to perform a task) or *procedural* information (specific information on how to complete routine aspects of a task) [27]. With scaffolding, the learner can perform more complex tasks or perform tasks more independently [27–29]. Scaffolding in general has been shown to improve learning outcomes in GBL [30]. However, superfluous scaffolding will increase extraneous cognitive load, for example by causing the learner to cross-reference provided instructions with information already present in their long-term memory, while insufficient or unnecessary scaffolding fails to lower the burden placed on the learner’s working memory, impeding the learning process in both situations [7, 31]. Consequently, it is critical to provide contingent scaffolding: the right type and level of support at the appropriate time and rate.

### Adaptive scaffolding

To ensure contingent scaffolding in computer-based learning environments such as digital GBL, adaptivity may be used: the automatic adjustment of a system to input from the player’s characteristics and choices [32]. While nonadaptive systems exacerbate differences between individuals, adaptations that are responsive to individual differences have been proposed to improve the equality and diversity of educational opportunities [33]. Adaptivity improves learning in hypermedia environments [34]. In GBL, several studies have investigated adaptivity, demonstrating promising effects on skill acquisition [35–37]. However, not all studies demonstrate favourable results [38].

Appropriate adaptive scaffolding should be triggered by indicators that identify the learner’s need for support. These indicators may be obtained before, during, or after a learning task. Examples include the learner's current knowledge level, cognitive load, stress measurements, performance assessments, or interaction traces documenting in-game events, choices, and behaviors, either separately or in combination [9, 10, 32, 39, 40]. Of these options, interaction traces in particular offer the advantage of unobtrusive and real-time collection, allowing for adaptations on a small timescale with short feedback loops. Examples of traces that can be used as indicators of performance in GBL include accuracy, speed, systematicity, and self-monitoring actions [41–44].

From the analysis presented above, we assume that adaptive scaffolding based on interaction traces is likely to positively influence cognitive load and improve learning task performance by freeing up working memory resources. In addition, this mechanism may improve the learner’s ability to self-regulate their learning, increase the transfer of learning, and influence learner engagement. We will discuss each of these below.

First, self-regulation of learning (SRL) refers to the modulation of affective, cognitive, and behavioral processes throughout a learning experience to reach the desired level of achievement [45]. Improved SRL can facilitate the learning of complex skills [46–51]. For example, students with higher developed SRL skills are better able to monitor their learning process during a task, recognize points of improvement, and use cognitive resources to support their learning, including help-seeking. Accordingly, SRL skills have been associated with improved confidence in learning, academic achievement, and success in clinical skills [47, 49, 52, 53]. SRL is especially important in GBL, as the inherent openness of the learning environment requires students to take control of their learning [54]. Several authors have presented suggestions on how to integrate CLT and SRL theory, arguing that metacognitive and self-regulatory demands should be conceptualized as a form of working memory load that can add to the cognitive load related to task performance [55–57]. In this light, optimizing cognitive load through adaptive scaffolding allows more resources for SRL activities. Indeed, adaptive scaffolding has been shown to improve self-regulated learning in non-game environments [8, 34, 38] and it has been suggested that adaptive scaffolding can prompt students to consciously regulate their learning [7].

Second, we expect adaptive scaffolding to influence the transfer of learning: applying one’s prior knowledge or skill to novel tasks, contexts, or related materials [58]. In GBL transfer may not arise naturally, as learning takes place in an environment that can be notably different from real-life practice. However, well-designed simulations and games are favorable for situated learning, which is known to improve learning and transfer [59]. Transfer can be promoted by effortful learning conditions that trigger active and deep processing. Instructional strategies aiming to create these conditions include variability in practice and encouraging elaboration. From the CLT perspective, these strategies aim to increase germane cognitive load. Adaptive scaffolding can enhance this process by decreasing extraneous load when the learner is overloaded and increasing germane load in the case of cognitive underload. Research demonstrating these effects is scarce, with a notable paper by Basu, Biswas [60] reporting improved transfer of computational thinking skills in students who received adaptive scaffolding during training.

Third, scaffolding is likely to influence game engagement, meaning the experience of being fully involved in an activity. The ease of starting, playing, and progressing in the game are important factors that influence engagement [61]. Engagement improves learning and increases information retention [62]. Different effects of scaffolding on engagement in GBL have been reported. For example, Barzilai and Blau [63] found no effect on engagement, while others have demonstrated decreases in engagement (e.g. [63–65]. It should be noted that these findings relate to nonadaptive scaffolding. If this scaffolding fails to optimize cognitive load, it is likely that learners will lose motivation to continue working on a task [66] and be less engaged. On the contrary, adaptive scaffolding designed to optimize cognitive load may positively influence engagement, as observed in one study by Chen, Law and Huang [7].

### Evaluating adaptive interventions

To specifically evaluate the effects of adaptive scaffolding, a yoked control research design may be applied [9, 35, 40]. In this design, matched participants are yoked (joined together) by receiving exactly the same treatment or interventions. From each pair, at random one participant is assigned to the *adaptive* condition and receives scaffolding tailored to their needs while their counterpart, assigned to the *nonadaptive* condition, is exposed to exactly the same scaffolding. Consequently, for the participant in the nonadaptive condition, the scaffolding is not intentionally adapted to their needs. The advantage of the yoked control design is that it allows the evaluation of the adaptation specifically. A difference in outcome may be attributed to the adaptation rather than the received support. However, depending on the heterogeneity in input used for the adaptive scaffolding, the nonadaptive scaffolding may coincidentally match the needs of the participant if their needs are the same as their counterpart adaptive in the adaptive condition. We will refer to the situation where participants in the nonadaptive condition coincidentally receive needed scaffolding as *tailored* scaffolding and the situation where they do not receive needed support as *nontailored* scaffolding.

### Purpose of the study

In the present study, we will investigate the effects of adaptive scaffolding in a medical emergency simulation game. We hypothesize that adaptive scaffolding will result in lower cognitive load through a decrease in extraneous cognitive load (hypothesis 1). This decrease in cognitive load will free up working memory capacity, allowing the learner to better process the information in the learning task. This will result in improved learning task performance (hypothesis 2) during gameplay, measured as accuracy (hypothesis 2a), speed (hypothesis 2b), and systematicity (hypothesis 2c). Working memory capacity may also be used for self-regulatory activities, including (more) self-monitoring (hypothesis 3a) and (more) help-seeking (hypothesis 3b). We hypothesize that improved task performance and self-regulation will lead to more effective learning, measured as improved transfer test performance (hypothesis 4). Regarding engagement, we hypothesize that adaptive scaffolding will improve learner engagement (hypothesis 5). In the current study, we will compare the adaptive and nonadaptive scaffolding groups for each hypothesis, as well as discuss post hoc exploratory analyses regarding the influence of tailored scaffolding in the non-adaptive group.

## Methods

### Design

To specifically evaluate the effects of adaptive scaffolding, we used a yoked control design as described above. Participants from the same university and either the same or immediately adjacent emergency care experience (0 cases, 1–2 cases, 3–5 cases) were matched in pairs. From each pair, one participant was randomly assigned to the adaptive scaffolding condition and the other to the nonadaptive condition. Ethical approval was provided by the Ethical Review Board of the Netherlands Association for Medical Education (dossier number 2021.3.5). Participants signed informed consent.

### Participants

#### Materials

##### Demographics questionnaire

A questionnaire was available regarding age, gender, study year, university of enrollment, and experience in emergency care. The questionnaire can be found in Appendix 1.

##### E-learning and knowledge test

In emergency care, healthcare professionals are trained to adhere to the ABCDE approach. This is an internationally used method in which the acronym “ABCDE” guides healthcare providers to examine and treat patients in the following phases: Airway, Breathing, Circulation, Disability, and Exposure. Following the ABCDE structure ensures that the most life-threatening conditions are treated first. For example, in the ‘B’ phase, the healthcare provider focuses on the breathing by listening to the lungs, checking for blue discoloration of the skin (cyanosis), ordering a chest X-ray if necessary, and providing inhalation medication if needed.

To provide students with knowledge of the ABCDE approach, an e-learning module consisting of ± 90 screens of information, illustrations, interactive questions, and videos on emergency medicine and the ABCDE method was available online. To confirm sufficient knowledge, we used a validated knowledge test on the ABCDE approach developed using the Delphi method [67]. The test contained 29 multiple-choice items. We applied a pass rate of 60% to ensure an adequate knowledge level. The test could be re-taken an unlimited number of times.

##### The abcdeSIM simulation game

In the abcdeSIM simulation game, players must assess and treat a virtual patient in a simulated virtual emergency department [5]. For familiarization, a walk-through tutorial and a practice scenario are available. In the practice scenario, the patient is healthy and their condition does not deteriorate. The game contains different scenarios in which a patient presenting with a medical condition must be examined, diagnosed, and treated within 15 min. After completing a scenario, a score and feedback on interventions are displayed. The game score is generated by adding points for correct interventions and subtracting points for harmful interventions or overlooked necessary interventions. If all vital interventions are performed, a time bonus of one extra point per second remaining is awarded. The patient’s underlying condition determines the required interventions, which were established by a panel of content experts.

We used the practice scenario and six emergency scenarios in a fixed order as follows: practice, deep venous thrombosis, chronic obstructive pulmonary disease, gastrointestinal bleeding, acute myocardial infarction, sepsis caused by pneumonia, and anaphylactic shock. Complexity increases with subsequent scenarios, meaning the patient’s condition is more severe and requires more or more urgent interventions.

##### Scaffolding in the abcdeSIM game

To enable scaffolding in the abcdeSIM game, we implemented additional supportive information and procedural information as described by Faber, Dankbaar and van Merriënboer [68]. Both types of information can be toggled on and off separately, resulting in four possible scaffolding combinations: both supportive and procedural information provided, neither provided, only supportive information provided and only procedural information provided.

**Fig. 1:**
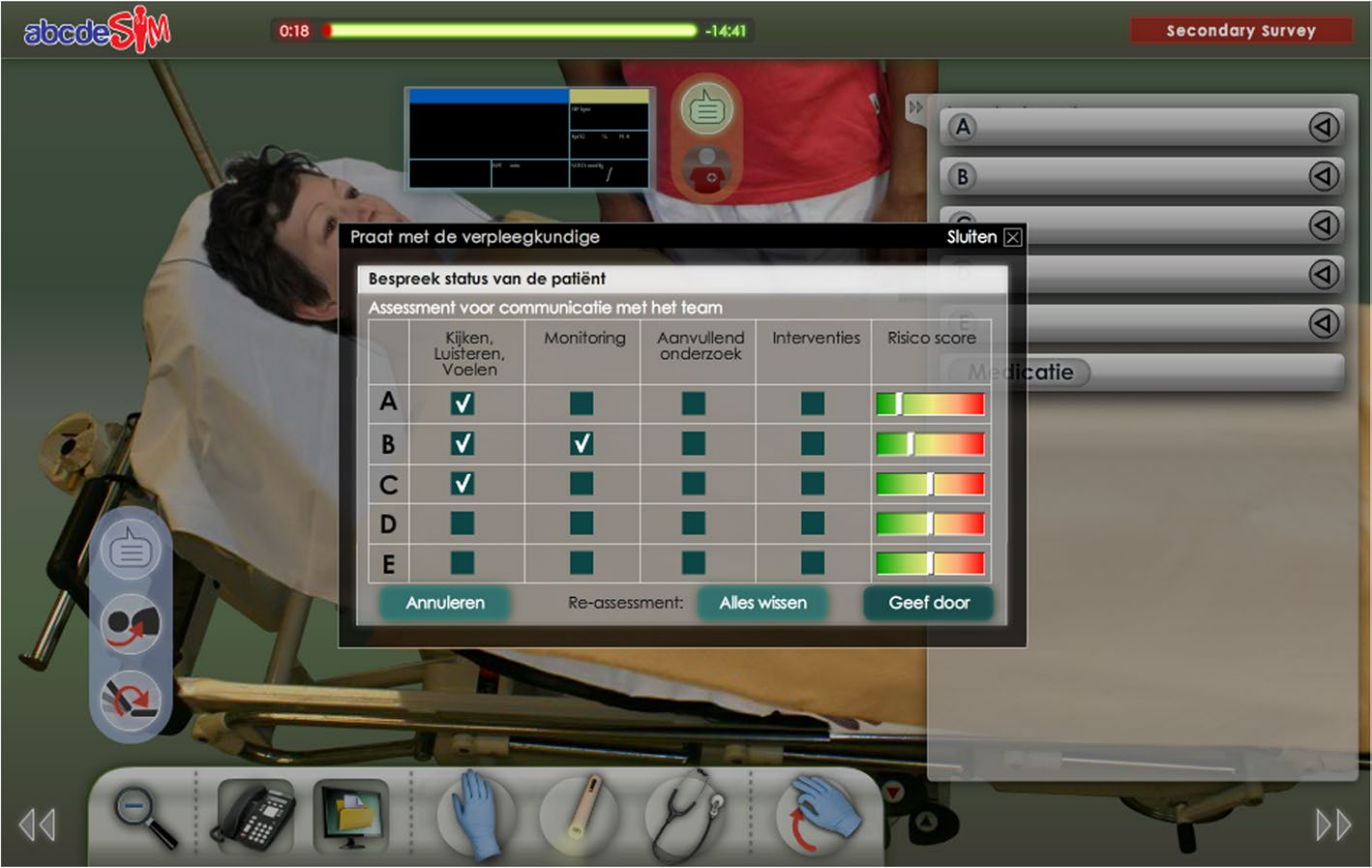
The basic checklist in abcdeSIM

**Fig. 2:**
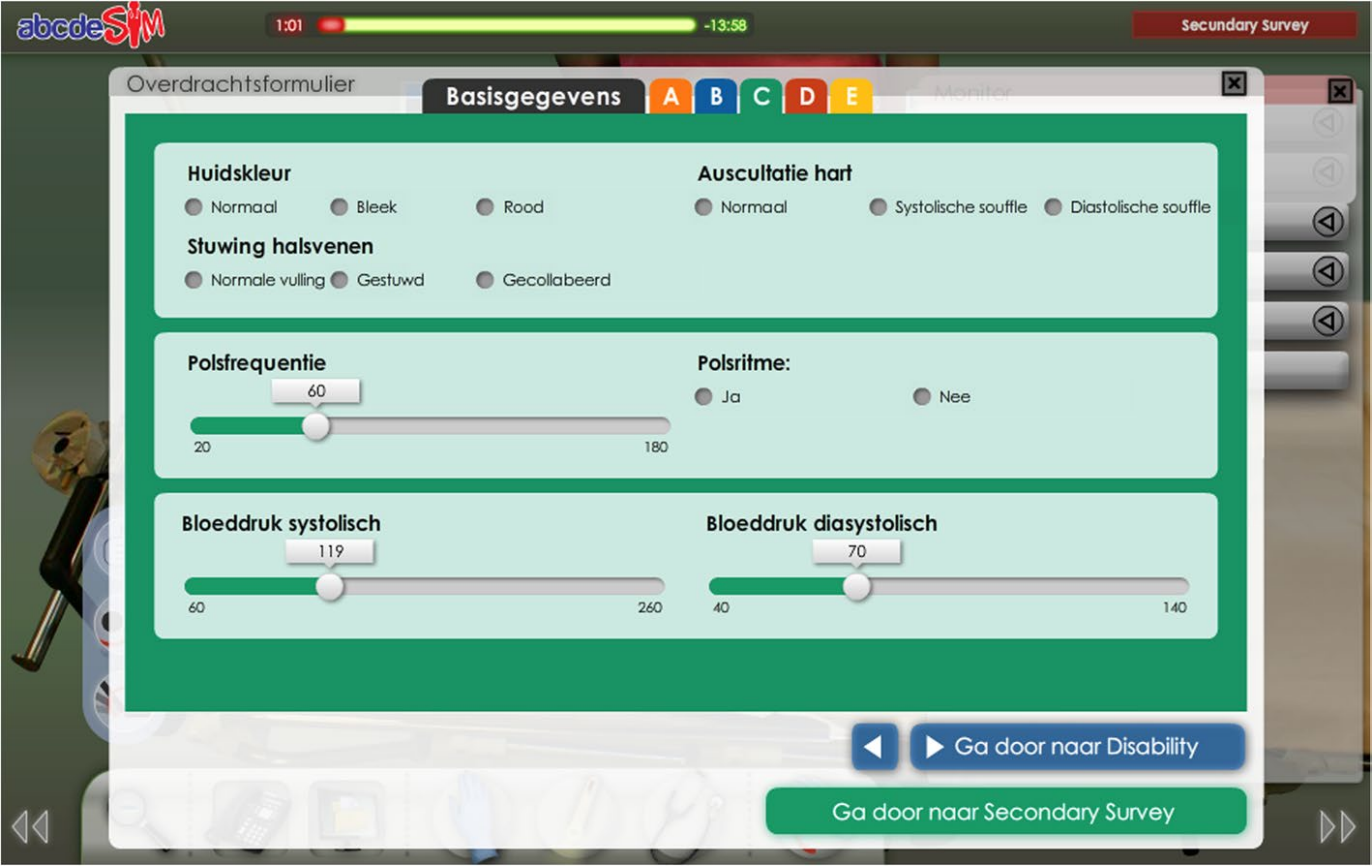
The extended checklist in abcdeSIM. A tab for general information (e.g. patient characteristics, presenting complaints) and one for each ABCDE phase prompt the player to examine specific features

Supportive information explains to the learners how a learning domain is organized and how to approach problems in that domain. It supports the learner in developing general schemas and problem-solving approaches [27]. In the abcdeSIM game, supportive information consisted of an extended checklist designed to facilitate the construction of a cognitive schema representing the ABCDE approach. The original abcdeSIM game includes a basic checklist intended to help the learner structure their approach (Fig. 1), consisting of simple checkboxes for the general approach in each ABCDE phase. However, it does not specify which actions or measurements should be performed. The extended checklist prompts the player to evaluate specific items in each phase, such as looking at skin color, listening to the heart, and measuring blood pressure in the ‘C’ phase (Fig. 2).

**Fig. 3:**
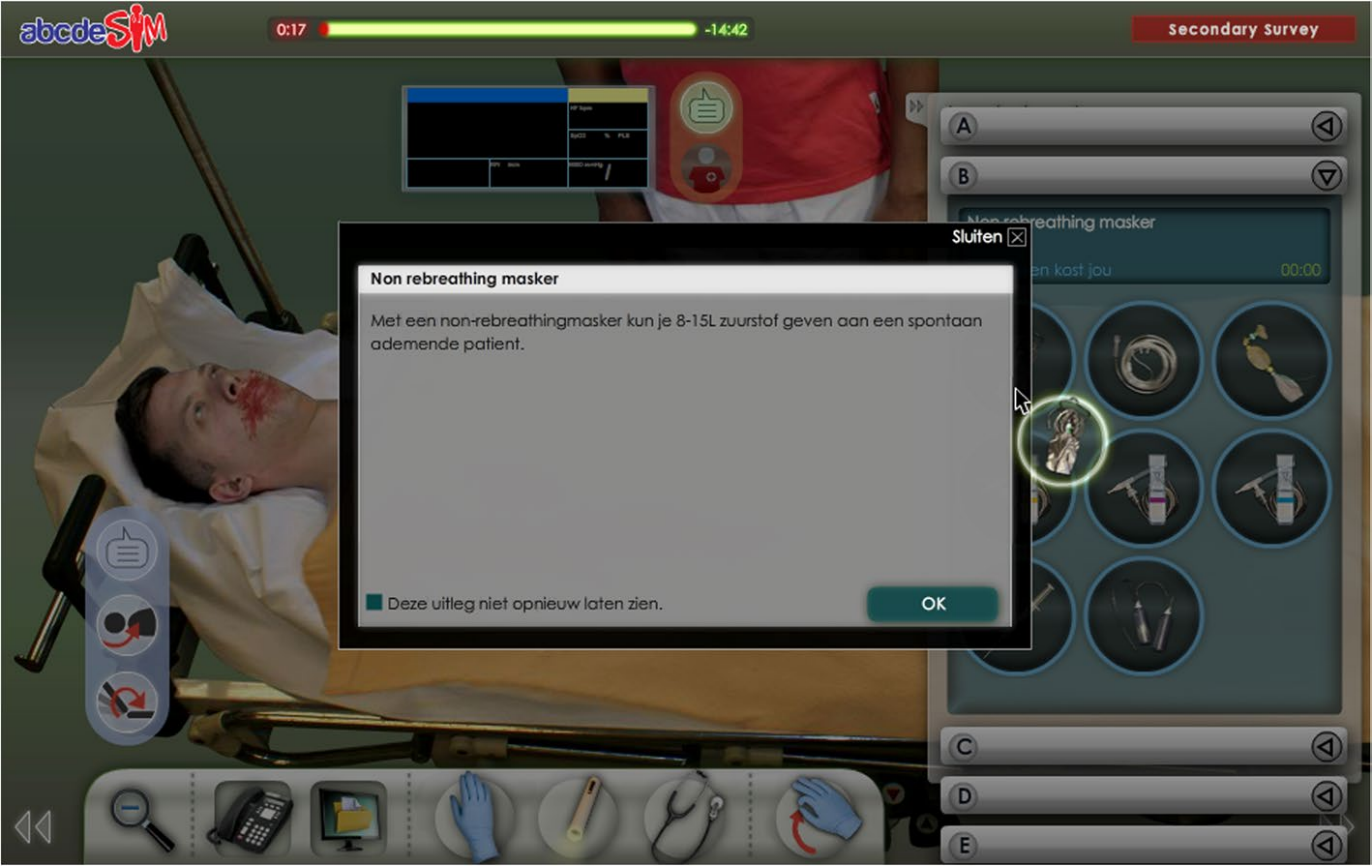
Tool information is provided in a dialogue box when a tool is selected. A checkbox in the bottom left corner enables the player to indicate they have read the information and do not want it to be shown again

Additional procedural information, meaning information provided in a just-in-time manner to complete routine aspects of tasks in the correct way [27], was implemented by showing a dialogue box upon tool selection. This dialogue box displays information on how and when to use the tool and appears every time the tool is selected until the player indicates to have read the information (Fig. 3).

##### Adaptive scaffolding algorithm

**Fig. 4:**
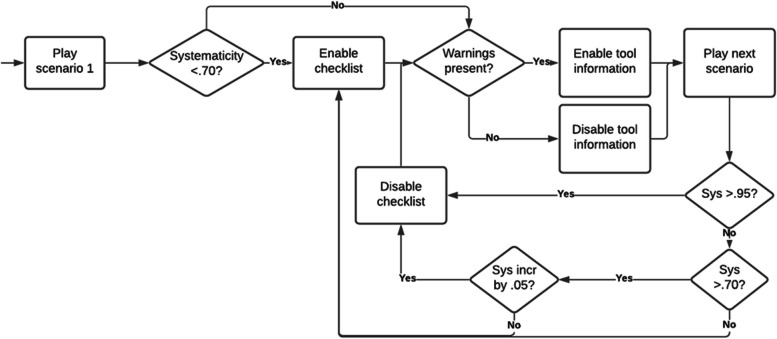
Algorithm for adaptive support

Adaptive scaffolding was provided based on different measures of task performance in the previously played scenario. The algorithm for adaptive scaffolding is summarized in Fig. 4. First, supportive information was provided when cognitive strategy use was deemed inadequate. We used systematicity in approach as a measure for adequate cognitive strategy use. Systematicity in approach, quantified using a Hidden Markov Model as described by Lee et al. [44], describes the level to which a player takes actions in the correct order. The model yields a score ranging from 0 to 1. A high systematicity indicates efficient knowledge-based cognitive strategies. To establish cutoff points for systematicity, we used data from a previous study with medical students playing the abcdeSIM game [41] (*M* = 0.71 and *SD* = 0.11). If the systematicity in the first scenario was below 0.70, additional supportive information was activated in the form of the extended checklist described above. For each subsequent scenario, the extended checklist was deactivated when systematicity increased at least 0.05 or was above 0.95, and activated if systematicity decreased by 0.05 or more.

Secondly, procedural information about tool use was provided based on the frequency of inappropriate tool use, quantified by counting the number of times the in-game nurse issued a warning to the player during a scenario. We consider this an indicator of insufficient procedural knowledge regarding the correct application of the instruments available in the game. The presence of any warnings led to additional procedural scaffolding by activating tool information for the subsequent scenario. If no warnings occurred, tool information was deactivated in the subsequent scenario.

### Outcome measures

#### Learning performance

To operationalize learning performance, meaning the performance in the game, we measured the accuracy of clinical decision-making, speed, and systematicity. *Accuracy* represents applied domain knowledge and was measured as the game score minus the time bonus. *Speed* represents the strength of cognitive strategies used and was shown to distinguish between experts and novices by Lee et al. [44]. We measured speed both as the total time to scenario completion and as the relative time to complete three critical interventions: introducing oneself, attaching the vital functions monitor, and providing oxygen. To allow comparison between different scenarios, *z*-scores were calculated per scenario after checking the normality of distribution. Finally, *systematicity* represents the quality of cognitive strategies, or how to approach unfamiliar problems in this context. We operationalized systematicity as a measure of how well the player adhered to the ABCDE approach, calculated as described under ‘Adaptive scaffolding algorithm’ above. An overview of all included outcome measures is provided in Table 1.

**Table 1:** Overview of outcome measures

	Indicative of	Derived from
Learning performance		
Accuracy	Applied domain knowledge	Points are added for correct interventions executed in the game and subtracted for harmful interventions. To attain a high score, the player must demonstrate knowledge of the interventions that are and are not indicated for this patients’ condition
Speed (absolute)	Strength of cognitive strategies	Total time to scenario completion
Speed (relative)	Strength of cognitive strategies	The relative time it has taken the player to perform critical actions required for all scenarios: attaching the monitor, applying oxygen, and introducing oneself to the patient
Systematicity in approach ^a^	Cognitive strategies – knowing how to approach an unfamiliar problem	Actions are labeled A, B, C, D, or E in chronological order. Using a Hidden Markov Model, a score ranging from 0 to 1 is calculated, indicating if the actions were performed in the appropriate order. The more the sequence resembles the correct order of A-B-C-D-E, the higher the score
Cognitive load		
Paas’ mental effort scale		Questionnaire asking how much mental effort they invested in the task on a 1–9 scale, labeled from 1 = ‘very, very low mental effort’ to 9 = ‘very, very high mental effort’
Self-regulation		
Self-monitoring		Number of times the player opens the checklist menu
Help-seeking		Number of times the player calls a medical specialist using the in-game telephone
Test performance		
Immediate test performance		Live scenario-based skill assessment of the ABCDE approach
Delayed test performance		Live scenario-based skill assessment of the ABCDE approach
Game engagement		Questionnaire on participants’ experience using the game

^a^Systematicity was used as a learning performance outcome measure and as input for the adaptive scaffolding algorithm

#### Cognitive load

Using an online questionnaire, we measured cognitive load for each game scenario using the Paas subjective rating scale [69] asking how much mental effort they invested in the task on a 1–9 scale, labeled from 1 = ‘very, very low mental effort’ to 9 = ‘very, very high mental effort’. According to Paas, Tuovinen [15], mental effort measured using this scale refers to “the aspect of cognitive load that is allocated to accommodate the demands imposed by the task” and as such may be considered to reflect the actual cognitive load.

#### Self-regulated learning

Interaction traces can offer insight into the use of specific SRL strategies in the game, such as monitoring, problem-solving, and decision-making processes [39, 70]. To quantify the use of specific SRL strategies, we recorded the number of times participants accessed the checklist as a measure of *monitoring* and the number of telephone calls to a medical specialist or consultant as a measure of *help-seeking*.

#### Transfer test performance

To quantify transfer test performance, we used a live scenario-based skill assessment of the ABCDE approach at two time points (immediate assessment and delayed assessment). Four different scenarios were designed by content experts to be distinct from the game scenarios and checked for similar complexity. The scenarios concerned patients presenting with hypoglycemia, urosepsis, pneumothorax, and ruptured aneurysm of the abdominal aorta. In the immediate assessment, participants were presented with first the hypoglycemia and then the urosepsis scenario. In the delayed assessment, they were presented with first the pneumothorax and then the ruptured aneurysm of the abdominal aorta scenario. Expert clinicians experienced in simulation-based training and assessment facilitated the scenarios, playing the role of nurse, and assessed the participants’ performance. A basic manikin and practice crash cart were used. Vital functions, patient responses, and additional information were provided by the scenario assessor. The participants did not have to perform psychomotor skills, such as placing an iv or attaching the monitor, but did have to indicate when to apply these skills. The assessor rated performance using an assessment instrument adapted from Dankbaar et al. [71]. The rating consisted of a Competency Scale (6 items on the ABCDE method and diagnostics, rated on a 7-point scale from 1 = “very weak” to 7 = “excellent”) and a Global Performance Scale using a single 10-point scale to rate ‘independent functioning in caring for acutely ill patients in the Emergency Department’ (10 = “perfect”) as if the participant were a recently graduated physician. The assessment instruments are shown in Appendix 2. To improve inter-rater reliability, the first author briefed all raters on the content of the scenarios, how to run the scenarios, how much support and guidance to provide during the assessment, and how to use the assessment instruments. Raters were blinded to the scaffolding conditions and the participant’s year of study. Feedback to the participant was provided only after the delayed assessment.

#### Game engagement

To measure game engagement, we used a questionnaire on participants’ experience adapted from Dankbaar, Stegers-Jager, Baarveld, Merrienboer, Norman, Rutten, et al. [5]. The questionnaire consists of 9 statements, including items such as: “I felt actively involved with the patient cases”, to be scored on a 5-point Likert scale (5 = fully agree). The questionnaire can be found in Appendix 3.

### Procedure

**Fig. 5:**
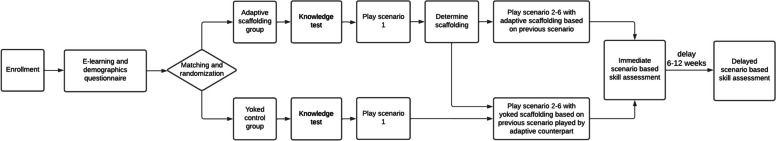
Study design

The overall study design is visualized in Fig. 5. After enrollment, all participants were given access to the e-learning module and completed the demographics survey. Next, they were randomly divided into matched pairs. After passing the knowledge test, participants gained online access to the six game scenarios.

In the scenarios, scaffolding was provided as follows:

1. *Adaptive scaffolding condition*: in the first patient scenario, no scaffolding was provided. In subsequent scenarios, adaptive scaffolding was provided as described above.
2. *Non-adaptive condition*: the yoked participant received the same scaffolding as the participant they were matched to. Each training sequence was allocated only once to one participant in the non-adaptive condition.

During the game scenarios, learning performance outcome measures were collected automatically. After each game scenario, participants were requested to indicate the cognitive load for the scenario in the separate online cognitive load questionnaire. After the sixth and final game scenario, they completed the engagement questionnaire. Within two weeks of completing the final game scenario, participants performed the first live scenario-based skill assessment. Six to twelve weeks later, participants returned for a delayed live scenario-based skill assessment to measure long-term retention. They could not access the abcdeSIM game between the two assessments.

### Analysis

#### Confirmatory analysis

For each game session, we used a specialized JavaScript parser to extract accuracy, scenario completion time, systematicity in approach, self-monitoring, and help-seeking as described by Faber, Dankbaar, Kickert, van den Broek and van Merriënboer [41]. The analysis was performed in R [72] using the Rstudio software version 1.2.1335 [73]. Data were visually inspected for normality. Differences between the groups in participant characteristics were tested for significance using paired *t*-tests for continuous variables and Stuart-Maxwell tests for categorical variables. We calculated Cronbach’s alpha for the questionnaires and assessment instruments to evaluate reliability. Multilevel correlations between the learning performance outcome measures were calculated using the correlation package [74].

For hypotheses 1, 2 and 3, we used multilevel regression (also known as linear mixed) models, taking into account the number of scenarios already played by the student. This type of model has been widely used in longitudinal data where repeated measurements of the same participants are taken over the study period [75]. We fitted a partially crossed linear mixed model, using the lme4 package [76]. We fit separate models for the following outcome measures: cognitive load (H1), accuracy, time spent on the scenario, time to vital interventions, and systematicity (H2), and frequency of self-monitoring and help-seeking (H3). We used the outcome measures as criterion measures and random intercepts for pair and participant as random effects, to account for the dependent data structure. As fixed effects, we included the number of scenarios played and the scaffolding condition (adaptive vs. non-adaptive). To calculate* p* values, we performed likelihood ratio tests comparing the full model with the effects in question against the model without the effects in question. Model comparisons can be found in Supplementary Table A. To test hypotheses 4 and 5, we performed a paired *t*-test for transfer test performance and engagement outcomes per condition.

#### Exploratory analysis

Because tailored scaffolding occurred, meaning participants in the nonadaptive group received the same support as they would have in the adaptive group, we performed separate exploratory subgroup analyses within the nonadaptive group. For learning performance, SRL, and cognitive load, we included these outcome measures as criterion measures and random intercepts for participants as random effects in multilevel regression models. As fixed effects, we included the number of scenarios played and whether supportive and procedural information was tailored. Model comparisons for the tailored scaffolding models can be found in Supplementary Table B. For test performance and engagement, we calculated Pearson’s *r* to test for correlations between the number of scenarios played with tailored scaffolding and the outcome measure.

## Results

### Baseline characteristics

Eighty-three medical students (age *M* = 22.8 years*, SD* = 1.8) participated in the study. One participant was excluded because they did not adhere to the study protocol. Sixty-nine participants completed all six game scenarios, resulting in 32 complete pairs. The other 19 participants either could not be matched or failed to complete the game scenarios.

Participants in the adaptive and nonadaptive groups were similar in age, gender, experience with emergency care, study year, and score on the knowledge test. Detailed characteristics are shown in Table 2. Tailored scaffolding was observed in 64.9% of the game scenarios played in the nonadaptive group, with an average of 3.9 tailored scenarios per participant (range 2–6). One participant in the nonadaptive group received tailored scaffolding on all six scenarios.

**Table 2:** Participant characteristics per group

	Adaptive (*N* = 31)	Non-adaptive (*N* = 31)	*p*-value
**Age**			0.570^a^
Mean (SD)	22.7 (1.901)	22.9 (1.672)	
Range	20.0—26.0	20.0—27.0	
**Gender**			0.606^b^
Male	8 (25.8%)	7 (22.6%)	
Female	23 (74.2%)	23 (74.2%)	
Other	0 (0.0%)	1 (3.2%)	
**Experience in emergency care**			0.171^b^
N-Miss	5	2	
0 cases	16 (61.5%)	14 (48.3%)	
1–2 cases	7 (26.9%)	9 (31.0%)	
3–5 cases	3 (11.5%)	6 (20.7%)	
**Study year**			0.884^a^
Mean (SD)	4.36 (1.082)	4.32 (1.045)	
Range	3.00—6.00	3.00—6.00	
**Score on knowledge test (%)**			0.158^a^
Mean (SD)	81.79 (10.00)	85.55 (8.03)	
Range	62.06—100.00	68.97 – 96.55	

^a^Paired t-test

^b^Stuart-Maxwell test

Sixty-four students matched in 32 pairs played a total of 384 game scenarios. The cognitive load questionnaire was completed for 244 game sessions played by 49 participants in 30 pairs (64.7% of game sessions). For seven game scenarios data were not available for analysis due to technical problems, resulting in data available for analysis for 377 game sessions played by 63 participants in 32 pairs for learning performance (accuracy, scenario completion time, and systematicity) and self-regulated learning (help-seeking and monitoring). Time to vital interventions could not be calculated in 160 sessions because one or more vital actions had been omitted, resulting in 221 sessions available for this analysis. Thirty student pairs completed the initial transfer test and twenty-three the delayed transfer test.

### Reliability of instruments

In contrast to previous research validating the knowledge test with acceptable internal consistency (Cronbach’s α = . 77, [67]) our data show poor consistency (α = 0.55, 95% CI [0.38—0.69]). Internal consistency for the assessment scores was excellent (α = 0.95, 95% CI [0.93—0.97]). There was a strong correlation between the score for the competency scale and the global performance scale, for both the immediate (r_p_ = 0.89,* p* < 0.001) and the delayed assessment (r_p_ = 0.90,* p* < 0.001).

A weak positive correlation was found between accuracy and total scenario time (r = 0.27, *p* = 0.015). For cognitive load, a significant correlation was present with systematicity (*r* = -0.28, *p* = 0.008) and total scenario time (*r* = 0.27, *p* = 0.015) but not accuracy, self-monitoring or help-seeking. Self-monitoring significantly correlated with accuracy (*r* = 0.32, *p* = 0.001) and total scenario time (*r* = 0.33, *p* < 0.001) but not with systematicity or help-seeking. For help-seeking we found a positive correlation with both accuracy (*r* = 0.35, *p* < 0.001) and total scenario time (*r* = 0.42, *p* < 0.001).

### Confirmatory analysis

#### Learning performance

Adaptive scaffolding condition did not significantly predict accuracy, time to vital interventions, and systematicity (Supplementary Table A). A trend toward longer scenario completion time was found for the adaptive scaffolding condition (β = 52.60 s, *SE* = 27.71, 95% CI = [-1.89 – 107.09], Supplementary Table B).

#### Cognitive load

The model including scaffolding condition could not significantly predict cognitive load compared with the model without scaffolding condition (χ^2^ = 1.71, *df* = 1,* p* = 0.191, Supplementary Table A).

#### Self-regulated learning

Adaptive scaffolding condition predicted a non-significant increase in the frequency of self-monitoring (β = 0.65, *SE* = 0.35, 95% CI [-0.03 – 1.34], Supplementary Table B). Help-seeking was not predicted by scaffolding condition.

#### Transfer test performance

We did not find differences in initial test performance between the conditions on both competency and global performance (respectively t = 0.71, *df* = 29,* p* = 0.480 and t = 0.93, *df* = 29,* p* = 0.357). Similarly, there were no differences in test performance on the delayed test (respectively t = -0.97, *df* = 22,* p* = 0.341 and t = -0.96, *df* = 21,* p* = 0.350). Results are shown in Table 3.

**Table 3:** Test performance results for the adaptive and nonadaptive condition

	Adaptive (*N* = 31)	Nonadaptive (*N* = 31)	*p*-value (paired *t*-test)
**Test performance on the first transfer test**			
**Competency score**			.480
—N-Miss	0	1	
—Mean (SD)	4.516 (0.799)	4.350 (1.213)	
—Range	3.083—6.167	2.167—6.583	
**Global performance score**			.357
—N-Miss	0	1	
—Mean (SD)	5.903 (1.274)	5.583 (1.848)	
—Range	4.000—9.000	2.500—9.000	
**Test performance on delayed transfer test**			
**Competency score**			.341
—N-Miss	0	8	
—Mean (SD)	4.618 (0.667)	4.837 (0.879)	
—Range	3.083—5.500	2.333—5.833	
**Global performance score**			.350
—N-Miss	1	8	
—Mean (SD)	5.783 (1.284)	6.087 (1.451)	
—Range	2.500—8.500	2.000—8.000	
**Engagement**			
—N-Miss			.455
—Mean (SD)	3.881 (0.494)	3.785 (0.480)	
—Range	2.778—4.556	2.444—4.667	

#### Engagement

Engagement was not significantly different between the adaptive and nonadaptive groups (*t* = 0.75662, *df* = 29,* p* = 0.455).

### Exploratory analysis

Thirty-two students in the non-adaptive group played a total number of 192 game scenarios. One scenario was not available for analysis due to technical issues, resulting in data for 191 game scenarios available for accuracy, scenario completion time, systematicity, help-seeking and self-monitoring. For 111 scenarios the time to vital interventions could be calculated. For 110 sessions, cognitive load data were measured. In 168 scenarios (87.9%) tailored supportive information was provided, while tailored procedural information was provided in 142 scenarios (74%). Descriptive statistics by tailored supportive and procedural scaffolding is available in Supplementary Table G and Supplementary Table H.

#### Learning performance

**Fig. 6:**
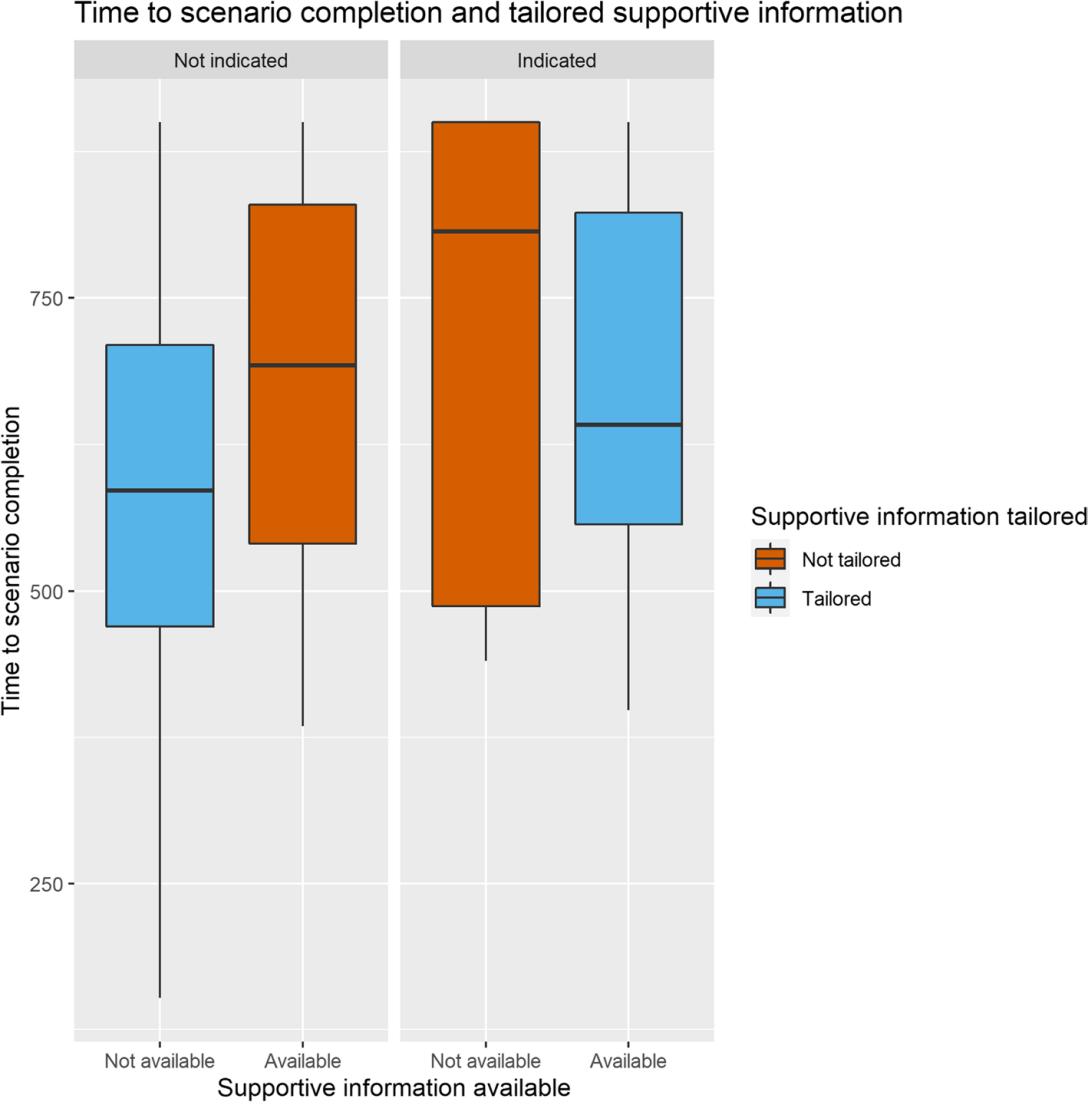
Scenario completion time and tailored supportive information. Participants receiving tailored supportive information (blue) are faster, compared to participants receiving nontailored supportive information (red). Left: participants who do not need supportive information are faster to complete the scenario when information is not provided (blue) compared to those who are provided with supportive information (red). Right: when supportive information is indicated, providing the information results in a faster completion (blue) compared to not providing supportive information (red)

Full model estimates can be found in Supplementary Table F. Tailored scaffolding significantly predicted scenario completion time (χ^2^ = 8.12, *df* = 2,* p* = 0.017) and time to vital interventions (χ^2^ = 8.54, *df* = 2,* p* = 0.014), but not accuracy and systematicity. As can be seen in Fig. 6, scenario completion time decreased both with tailored supportive and procedural information (respectively β = -90.57, *SE* = 35.35, 95% CI [-160.13 – -21.02] and β = -36.76, *SE* = 27.10, 95% CI [-90.23 – 16.72]). Tailored supportive information strongly decreased time to vital interventions (β = -0.82, *SE* = 0.32, 95% CI [-1.45 – -0.19]) while tailored procedural information had a weaker opposite effect, slowing the participants down (β = 0.32, *SE* = 0.25, 95% CI [-0.18 – 0.83]).

#### Cognitive load

Including tailored scaffolding significantly improved the model to predict cognitive load (χ^2^ = 14,85, *df* = 6,* p* = 0.021, Supplementary Table B). As shown in Fig. 5, tailored supportive information significantly lowered cognitive load (respectively β = -0.88, *SE* = 0.34, 95% CI [-1.56 – -0.20] Fig. 5) and a similar trend was observed for tailored procedural information (β = -0.51, *SE* = 0.30, 95% CI [-1.10 – 0.09]). Full results of the model can be found in Supplementary Table C.

#### Self-regulated learning

**Fig. 7:**
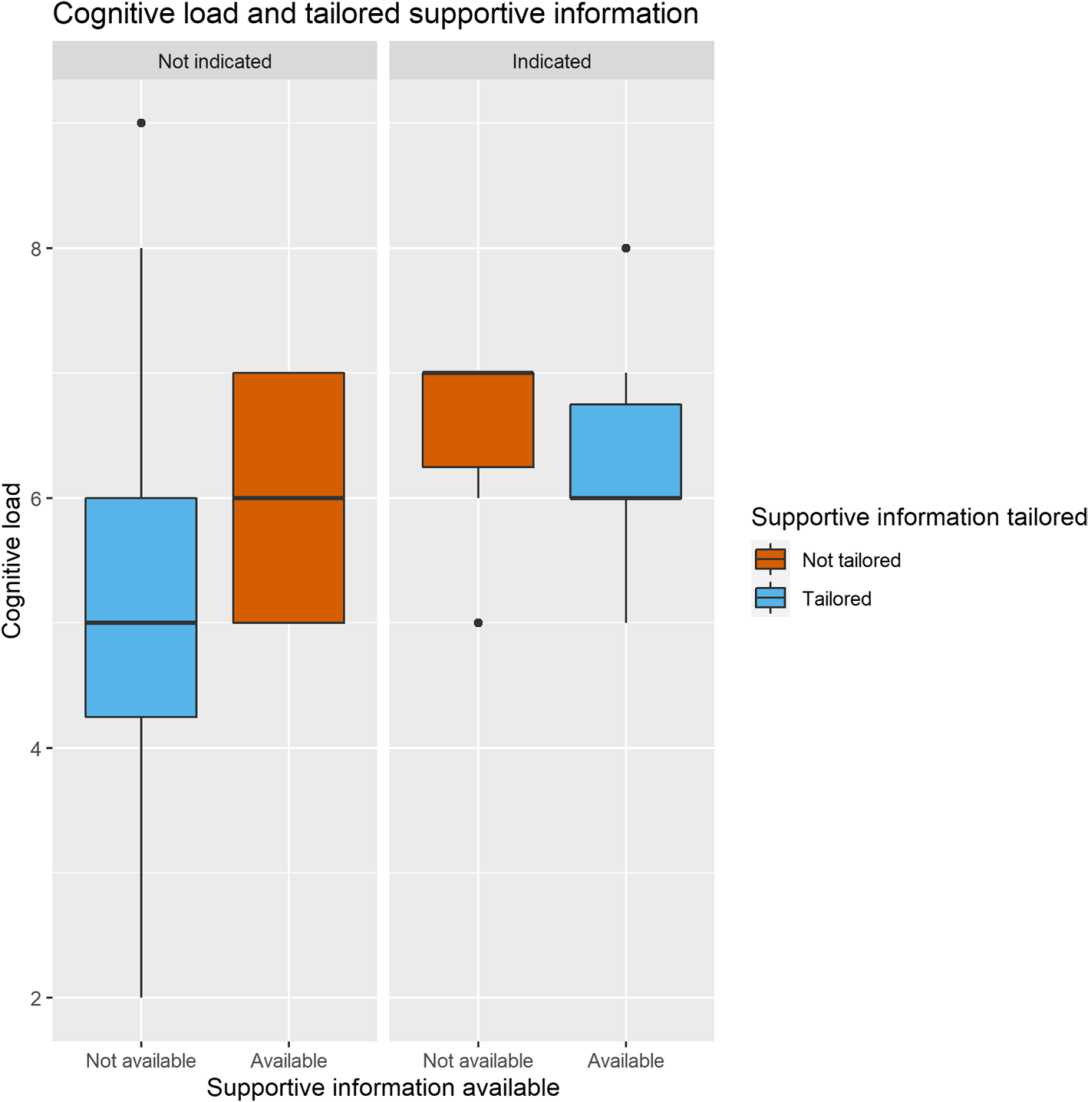
Cognitive load and tailored supportive information. Tailored supportive information (blue) results in a lower cognitive load compared with nontailored supportive information (red). Left: participants who do not need supportive information experience higher cognitive load when information is provided compared to those who are not provided with supportive information. Right: when supportive information is indicated, providing the information results in a lower cognitive load compared to not providing supportive information

**Fig. 8:**
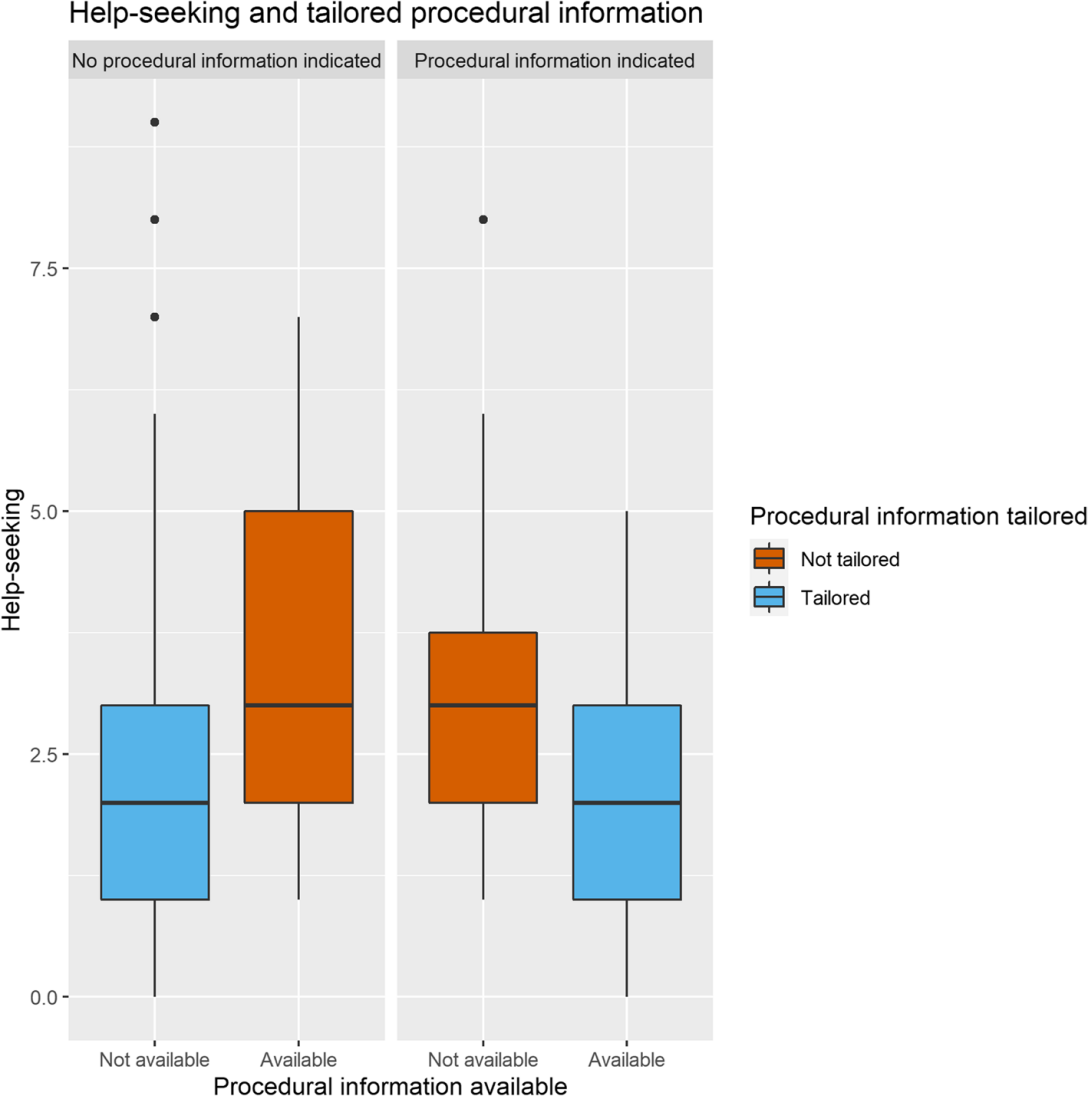
Help-seeking actions. Participants for whom procedural information is tailored (blue) seek help less often compared to participants for whom procedural information is not tailored (red)

**Fig. 9:**
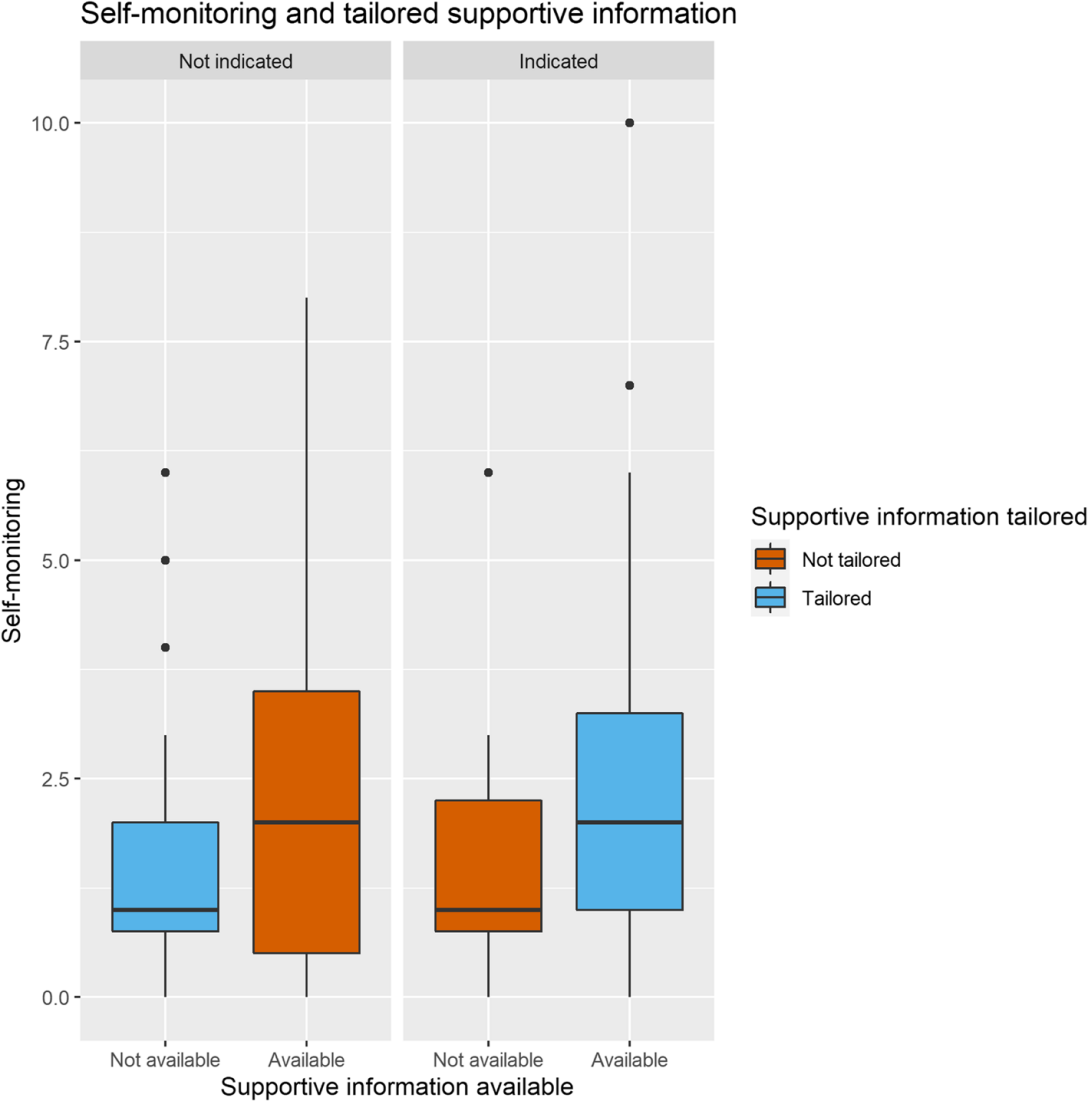
Self-monitoring behavior increases when supportive information is available, regardless of whether the information was tailored to the player’s behavior

In the nonadaptive group, tailored scaffolding significantly predicted both self-monitoring and help-seeking (respectively χ^2^ = 8.39, *df* = 2,* p* = 0.015 and χ^2^ = 6.99, *df* = 2,* p* = 0.030). Tailored supportive information decreased the frequency of self-monitoring in the scenario in which it was provided (β = -0.85, *SE* = 0.30, 95% CI [-1.44 – -0.26]) but had no large influence on help-seeking. In contrast, tailored procedural information did not influence self-monitoring significantly, but decreased help-seeking (β = -0.81, *SE* = 0.31, 95% CI [-1.41 – -0.21]), as can be seen in Fig. 7. Visual inspection (Figs. 8 and 9) suggests that the presence of the extended checklist increased monitoring behavior, regardless of the student’s needs. A post hoc multilevel model was constructed using self-monitoring as a criterion measure, random intercepts for participants, and as fixed effects the number of scenarios played, whether or not supportive and procedural information was available, and whether supportive and procedural information was tailored. This model was significantly different from the original model without the availability of supportive and procedural information (χ^2^ = 45.49, *df* = 2,* p* < 0.001) and showed that the presence of the extended checklist significantly increased self-monitoring (β = 1.52, *SE* = 0.21, 95% CI [1.11– 1.94]).

#### Transfer test performance

Looking at the influence of tailored scaffolding in the nonadaptive group, competency and global performance were not significantly correlated with the number of scenarios with tailored scaffolding on the first assessment (respectively r_p_ = 0.07, *p* 0.694 and r_p_ = -0.01,* p* = 0.944), and on the delayed assessment (r_p_ = -0.13,* p* = 0.537 and r_p_ = -0.10,* p* = 0.641).

#### Engagement

The number of scenarios with tailored scaffolding did not correlate with engagement in the non-adaptive group (r_p_ = 0.04,* p* = 0.838).

## Discussion

This study investigated the effects of adaptive scaffolding in a medical emergency simulation game on cognitive load, self-regulation, learning performance, transfer test performance, and engagement in a yoked control design. Apart from a trend towards more frequent self-monitoring and a longer time to scenario completion, we found no significant differences between the adaptive and nonadaptive groups. Unfortunately, the study’s power to detect differences between the groups was reduced because participants in the nonadaptive group also received scaffolding tailored to their needs in 64.9% of the game scenarios. This likely occurred because participants in both groups displayed comparable in-game behaviors. A similar limitation was mentioned by Salden, Paas and van Merriënboer [40], proposing that homogeneity in prior knowledge and expertise level explain this phenomenon, although they do not describe to what extent it occurred. Consequently, we performed exploratory analyses in the nonadaptive subgroup investigating the effects of tailored versus non-tailored scaffolding.

Regarding hypothesis 1, the results of the exploratory analyses suggest that tailored scaffolding lowered cognitive load. This effect can be explained by a reduction in extraneous load: students who do not require support do not need to cross-reference the information provided by the scaffolding with existing schemas, while students who lack knowledge on how to proceed are given scaffolding that can organize their learning [3].

Regarding learning performance (hypothesis 2), accuracy and systematicity could not be predicted and results regarding speed were mixed. While the adaptive group as a whole took longer to complete the scenarios compared with the nonadaptive group, in the nonadaptive group tailored scaffolding shortened the time to scenario completion. Time to vital interventions decreased with tailored supportive information but increased with tailored procedural information. In the literature, different effects from different types of scaffolds have been described (e.g., Wu and Looi [77]), with general prompts (similar to the supportive information used in this study) stimulating metacognitive activities, like self-monitoring, and specific prompts stimulating reflection on domain-related tasks and task-specific skills. Two explanations for our findings come to mind: first and foremost, reading the procedural information during task execution takes time by itself that immediately adds to the time to vital interventions. Secondly, the supportive information may stimulate learners to go back to the standard approach they have learned, helping them back on track.

Regarding self-monitoring (hypothesis 3a), in contrast to our findings comparing the adaptive and nonadaptive group, we found significantly reduced self-monitoring with tailored supportive information. This contrasts with previous research in non-game environments, where increases in self-regulation have been observed with adaptive scaffolding, either provided by human tutors [8, 78] or through rule-based artificial intelligence [38]. Visual inspection of our data and further exploratory post hoc analysis suggested that the presence of supportive information in itself increased the frequency of self-monitoring, while tailored scaffolding had no significant effects on self-monitoring frequency. This finding should be confirmed in an appropriately powered study, possibly combining interaction trace measures of SRL with other measures such as systematic observations [79], think-aloud protocols [80], micro-analytic questions [81], or eye-tracking data [82].

Help-seeking (hypothesis 3b) decreased with tailored procedural support. Participants who did not require procedural support and did not receive it, as well as those who did require procedural support and did receive it, sought help less often. Possibly, the tailored procedural information accurately provided the information the participants needed; hence the provision of help did not add much. We found no improvements in test performance (hypothesis 4) and learner engagement (hypothesis 5) with tailored scaffolding, likely because the analyses in the nonadaptive group had insufficient power for these single-timepoint outcomes.

Our study had several strengths. We included students from three different universities in a double-blinded randomized study design. The study intervention provided multiple scenarios and we measured performance on several dimensions, including transfer test performance and retention. To our knowledge, this study is the first one to investigate the effects of adaptive scaffolding on learning performance as well as transfer performance in the context of game-based learning. However, our findings must be interpreted in light of the following limitations.

The first limitation regards the occurrence of coincidental tailored scaffolding in the nonadaptive group. As described above, this reduced the study’s power in comparing adaptive and non-adaptive support. To avoid this, future research should attempt to increase the differences between the adaptive and nonadaptive groups. For example, a different sampling strategy aiming to increase heterogeneity would decrease the incidence of adaptive scaffolding. This could involve recruiting more expert learners (e.g. residents) as well as novices, and not matching the pairs by experience. Other options include implementing a larger number of unique input variables for the adaptive algorithm or applying a different research design. This design could incorporate an adaptive group, a control group that does not receive any scaffolding, and another group receiving random scaffolding. The second limitation concerns the application of the adaptive scaffolding in the next scenario, instead of providing the scaffolding in the scenario where the need for scaffolding was identified. The timing of scaffolding influences its effects. For example, study material provided before play has proven more effective than the other way around [63]. This may have attenuated the effects of the scaffolding provided in our study.

A final limitation in our study was the use of a single-item measure for cognitive load. We chose the Paas single item mental effort scale because it is sensitive to small changes [83, 84], easy to use and barely interrupts gameplay. However, we failed to/did not find significant correlations between cognitive load and self-regulatory activities although we expected increases in germane load. A differentiated cognitive load measure could provide more insight into how adaptive scaffolding increases germane load, meaning the active resources invested by the learner, compared with the load produced by the task itself, consisting of intrinsic and extraneous load. Apart from the previously mentioned 10-item scale by Leppink et al. [16], the 8-item questionnaire by Klepsch and Seufert [85] and the 15-item scale developed by Krieglstein et al. [86] appear promising instruments that distinguish between active and passive mental load. Challenges in using these questionnaires involve the larger number of items, interrupting game flow, as well as the limited reliability for measuring germane cognitive load and sensitivity to changes in item formulation that may be necessary for translation. As germane cognitive load is dependent on intrinsic cognitive load [87, 88], adding physiological measures (see Ayres et al. [21]) to non-intrusively provide insight into intrinsic cognitive load may help clarify the role of scaffolding in relation to task complexity.

## Conclusions

We could not find evidence to support our hypothesis of improved performance and lower cognitive load in adaptive scaffolding in game-based learning. Exploratory analyses do suggest a possible effect of tailored scaffolding. To further build on these findings, we offer three recommendations for research in adaptive scaffolding in game-based learning/GBL?. First, researchers should choose their research design and adaptive algorithm carefully to prevent coincidental adaptive scaffolding in the control group, as described above. Secondly, we recommend a more granular approach to measuring cognitive load, combining multi-item subjective measurements with physiological measurements. Finally, the specific effects of adaptive scaffolding should be investigated, including different effects for various types of adaptive scaffolding. Options include incorporating eye tracking, think-aloud protocols, or cued recall interviews to elucidate the mechanisms through which adaptive scaffolding influenced self-regulation in the game.

Tailored scaffolding shows promise as a technique to optimize cognitive load in GBL. When designing an adaptive GBL or computer-based simulation environment, we recommend that educators and developers work towards adaptive scaffolding as a team from the start. This will facilitate the establishment of reliable indicators of performance, self-regulation, and learning, as well as the design of appropriate, preferably real-time, scaffolding. For educators or developers who are unable to implement adaptive scaffolding, supportive information may be provided as a static scaffold to improve self-monitoring.

To conclude, this study into the effects of scaffolding in a medical emergency simulation game suggests that implementing tailored scaffolding in GBL may optimize cognitive load. Tailored supportive and procedural information have different effects on self-regulation and learning performance, necessitating further research into the effects of adaptive support as well as the design of well-calibrated algorithms. Considering the pivotal role of cognitive load in learning, these findings should inform instructional design both in game-based learning as well as other educational formats.

### Supplementary Information

Supplementary Material 1.

Supplementary Material 2.

## Data Availability

The datasets used during the current study are available from the corresponding author on reasonable request.

## Abbreviations

ABCDE: Airway, breathing, circulation, disability, exposure: a mnemonic used in emergency medicine
CLT: Cognitive load theory
GBL: Game-based learning
SRL: Self-regulated learning or self-regulation of learning

## References

[1] Abdulmajed H, Park YS, Tekian A. Assessment of educational games for health professions: a systematic review of trends and outcomes. 2015. 10.3109/0142159X.2015.1006609 25803590

[2] de Freitas S. Are games effective learning tools? A review of educational games. J Educ Technol Soc. 2018;21(2):74–84.

[3] Kalyuga S, Plass JL. Evaluating and managing cognitive load in games. In: Ferdig RE, editor. Handbook of Research on Effective Electronic Gaming in Education. Hershey, PA: IGI Global; 2009. p. 719–37.

[4] Dankbaar MEW, Alsma J, Jansen EEH, van Merrienboer JJG, van Saase JLCM, Schuit SCE. An experimental study on the effects of a simulation game on students’ clinical cognitive skills and motivation. Adv Health Sci Educ Theory Pract. 2016;21(3):505–21. 26433730 10.1007/s10459-015-9641-x PMC4923100

[5] Dankbaar MEW, Roozeboom MB, Oprins EAPB, Rutten F, van Merrienboer JJG, van Saase JLCM, et al. Preparing residents effectively in emergency skills training with a serious game. Simul Healthc. 2017;12(1):9–16. 27764018 10.1097/SIH.0000000000000194 PMC5291282

[6] Munshi A, Biswas G, Baker R, Ocumpaugh J, Hutt S, Paquette L. Analysing adaptive scaffolds that help students develop self-regulated learning behaviours. J Comput Assist Learn. 2023;39(2):351–68. 10.1111/jcal.12761.

[7] Chen C-H, Law V, Huang K. Adaptive scaffolding and engagement in digital game-based learning. Educ Technol Res Dev. 2023;71:1785–98. 10.1007/s11423-023-10244-x

[8] Azevedo R, Cromley JG, Seibert D. Does adaptive scaffolding facilitate students’ ability to regulate their learning with hypermedia? Contemp Educ Psychol. 2004;29(3):344–70. 10.1016/j.cedpsych.2003.09.002

[9] Kalyuga S, Sweller J. Rapid dynamic assessment of expertise to improve the efficiency of adaptive e-learning. Educ Tech Res Dev. 2005;53(3):83–93. 10.1007/BF02504800

[10] Hennings C, Ahmad M, Lohan K. Real-Time Adaptive Game to Reduce Cognitive Load. In: Proceedings of the 9th International Conference on Human-Agent Interaction (HAI '21). New York: Association for Computing Machinery; 2021. p. 342–7. https://doi-org.ru.idm.oclc.org/10.1145/3472307.3484674.

[11] Ke F. Designing and integrating purposeful learning in game play: a systematic review. Educ Tech Res Dev. 2016;64(2):219–44. 10.1007/s11423-015-9418-1

[12] Sweller J, van Merriënboer JJG, Paas F. Cognitive architecture and instructional design: 20 years later. Educ Psychol Rev. 2019;31(2):261–92. 10.1007/s10648-019-09465-5

[13] Sweller J, et al. Cognitive Load Theory. New York: Springer; 2011. 10.1007/978-1-4419-8126-4.

[14] Kalyuga S, Ayres P, Chandler P, Sweller J. The expertise reversal effect. Educ Psychol. 2003;38(1):23–31. 10.1207/S15326985EP3801_4

[15] Paas F, Tuovinen, JE, Tabbers, H, Van Gerven, PWM. Cognitive Load Measurement as a Means to Advance Cognitive Load Theory. Educ Psychol. 2003;38(1):63–71. 10.1207/S15326985EP3801_8.

[16] Leppink J, Paas F, van der Vleuten CPM, van Gog T, van Merriënboer JJG. Development of an instrument for measuring different types of cognitive load. Behav Res Methods. 2013;45(4):1058–72. 23572251 10.3758/s13428-013-0334-1

[17] Hart SG, Staveland, LE. Development of NASA-TLX (Task Load Index): Results of Empirical and Theoretical Research. Adv Psychol. 1988;52:139-83. P. A. Hancock and N. Meshkati, Amsterdam

[18] Brünken R, Steinbacher S, Plass JL, Leutner D. Assessment of cognitive load in multimedia learning using dual-task methodology. Exp Psychol. 2002;49(2):109. 12053529 10.1027//1618-3169.49.2.109

[19] Rojas D, Haji F, Shewaga R, Kapralos B, Dubrowski A. The impact of secondary-task type on the sensitivity of reaction-time based measurement of cognitive load for novices learning surgical skills using simulation. Stud Health Technol Inform. 2014;196:353–9. 24732535

[20] Leppink J. Cognitive load measures mainly have meaning when they are combined with learning outcome measures. Med Educ. 2016;50(9):979-. 27562897 10.1111/medu.13126

[21] Ayres P, Lee JY, Paas F, van Merriënboer JJG. The Validity of Physiological Measures to Identify Differences in Intrinsic Cognitive Load. Front Psychol. 2021;12:702538. 34566780 10.3389/fpsyg.2021.702538 PMC8461231

[22] Skulmowski A, Rey GD. Measuring cognitive load in embodied learning settings. Front Psychol. 2017;8:1191. 28824473 10.3389/fpsyg.2017.01191 PMC5539229

[23] van Merriënboer JJG, Kester L. The Four-Component Instructional Design Model: Multimedia Principles in Environments for Complex Learning. The Cambridge handbook of multimedia learning. New York: Cambridge University Press; 2005. p. 71–93.

[24] van Merriënboer JJ, Sweller J. Cognitive load theory in health professional education: design principles and strategies. Med Educ. 2010;44(1):85–93. 20078759 10.1111/j.1365-2923.2009.03498.x

[25] Könings KD, van Zundert M, van Merriënboer JJG. Scaffolding peer-assessment skills: Risk of interference with learning domain-specific skills? Learn Instr. 2019;60:85–94. 10.1016/j.learninstruc.2018.11.007

[26] Vygotsky LS. Mind in Society: Development of Higher Psychological Processes. Edited by Michael Cole, Vera Jolm-Steiner, Sylvia Scribner, and Ellen Souberman. Cambridge: Harvard University Press; 1978. 10.2307/j.ctvjf9vz4. Original manuscripts [ca. 1930–1934].

[27] van Merriënboer JJG, Kirschner PA. Ten steps to complex learning : a systematic approach to four-component instructional design. 3rd ed. London: Routledge; 2017. p. 399.

[28] Merrill MD. A task-centered instructional strategy. J Res Technol Educ. 2007;40(1):5–22. 10.1080/15391523.2007.10782493

[29] Puntambekar S, Hubscher R. Tools for scaffolding students in a complex learning environment: what have we gained and what have we missed? Educ Psychol. 2005;40(1):1–12. 10.1207/s15326985ep4001_1

[30] Cai Z, Mao P, Wang D, He J, Chen X, Fan X. Effects of scaffolding in digital game-based learning on student’s achievement: a three-level meta-analysis. Educ Psychol Rev. 2022;34(2):537–74. 10.1007/s10648-021-09655-0

[31] van de Pol J, Volman M, Oort F, Beishuizen J. The effects of scaffolding in the classroom: support contingency and student independent working time in relation to student achievement, task effort and appreciation of support. Instr Sci. 2015;43(5):615–41. 10.1007/s11251-015-9351-z

[32] Streicher A, Smeddinck JD. Personalized and Adaptive Serious Games. In: Dörner R, Göbel S, Kickmeier-Rust M, Masuch M, Zweig K, editors. Entertainment Computing and Serious Games: International GI-Dagstuhl Seminar 15283, Dagstuhl Castle, Germany, July 5–10, 2015, Revised Selected Papers. Cham: Springer International Publishing; 2016. p. 332–77.

[33] Snow RE. Individual differences and the design of educational programs. Am Psychol. 1986;41(10):1029–39. 10.1037/0003-066X.41.10.1029

[34] Azevedo R, Cromley J, Moos D, Greene J, Winters F. Adaptive Content and Process Scaffolding: a key to facilitating students’ self-regulated learning with hypermedia. Psychol Test Assess Model. 2011;53:106.

[35] Corbalan G, Kester L, van Merriënboer JJG. Selecting learning tasks: Effects of adaptation and shared control on learning efficiency and task involvement. Contemp Educ Psychol. 2008;33:733–56. 10.1016/j.cedpsych.2008.02.003

[36] Leutner D. Guided discovery learning with computer-based simulation games: Effects of adaptive and non-adaptive instructional support. Learn Instr. 1993;3(2):113–32. 10.1016/0959-4752(93)90011-N

[37] Serge SR, Priest HA, Durlach PJ, Johnson CI. The effects of static and adaptive performance feedback in game-based training. Comput Hum Behav. 2013;29(3):1150–8. 10.1016/j.chb.2012.10.007

[38] Lim L, Bannert M, van der Graaf J, Singh S, Fan Y, Surendrannair S, et al. Effects of real-time analytics-based personalized scaffolds on students’ self-regulated learning. Comput Hum Behav. 2023;139: 107547. 10.1016/j.chb.2022.107547

[39] Serrano-Laguna Á, Manero B, Freire M, Fernández-Manjón B. A methodology for assessing the effectiveness of serious games and for inferring player learning outcomes. Multimed Tools Appl. 2018;77(2):2849–71. 10.1007/s11042-017-4467-6

[40] Salden RJCM, Paas F, van Merriënboer JJG. Personalised adaptive task selection in air traffic control: Effects on training efficiency and transfer. Learn Instr. 2006;16(4):350–62. 10.1016/j.learninstruc.2006.07.007

[41] Faber TJE, Dankbaar MEW, Kickert R, van den Broek WW, van Merriënboer JJG. Identifying indicators to guide adaptive scaffolding in games. Learn Instr. 2022;83:101666. 10.1016/j.learninstruc.2022.101666

[42] Kang J, Liu M, Qu W. Using gameplay data to examine learning behavior patterns in a serious game. Comput Hum Behav. 2017;72:757–70. 10.1016/j.chb.2016.09.062

[43] Riemer V, Schrader C. Impacts of behavioral engagement and self-monitoring on the development of mental models through serious games: Inferences from in-game measures. Comput Hum Behav. 2016;64:264–73. 10.1016/j.chb.2016.06.057

[44] Lee JY, Donkers J, Jarodzka H, van Merriënboer JJG. How prior knowledge affects problem-solving performance in a medical simulation game: Using game-logs and eye-tracking. Comput Hum Behav. 2019;99:268–77. 10.1016/j.chb.2019.05.035

[45] Karoly P. Mechanisms of self-regulation: a systems view. Annu Rev Psychol. 1993;44(1):23–52. 10.1146/annurev.ps.44.020193.000323

[46] van Houten-Schat MA, Berkhout JJ, van Dijk N, Endedijk MD, Jaarsma ADC, Diemers AD. Self-regulated learning in the clinical context: a systematic review. Med Educ. 2018;52(10):1008–15. 29943415 10.1111/medu.13615 PMC6175376

[47] Cho KK, Marjadi B, Langendyk V, Hu W. The self-regulated learning of medical students in the clinical environment - a scoping review. BMC Med Educ. 2017;17(1):112-. 28693468 10.1186/s12909-017-0956-6 PMC5504849

[48] Brydges R, Manzone J, Shanks D, Hatala R, Hamstra SJ, Zendejas B, et al. Self-regulated learning in simulation-based training: A systematic review and meta-analysis. Med Educ. 2015;49(4):368–78. 25800297 10.1111/medu.12649

[49] Sabourin JL, Shores LR, Mott BW, Lester JC. Understanding and predicting student self-regulated learning strategies in game-based learning environments. Int J Artif Intell Educ. 2013;23(1–4):94–114. 10.1007/s40593-013-0004-6

[50] Boekaerts M, Minnaert A. Self-regulation with respect to informal learning. Int J Educ Res. 1999;31:533–44. 10.1016/S0883-0355(99)00020-8

[51] de Bruin ABH, van Merriënboer JJG. Bridging Cognitive Load and Self-Regulated Learning Research: a complementary approach to contemporary issues in educational research. Learn Instr. 2017;51:1–9. 10.1016/j.learninstruc.2017.06.001

[52] Cleary TJ, Durning SJ, Artino AR. Microanalytic assessment of self-regulated learning during clinical reasoning tasks. Acad Med. 2016;91(11):1516–21. 27191840 10.1097/ACM.0000000000001228

[53] Nietfeld JL, Shores LR, Hoffmann KF. Self-regulation and gender within a game-based learning environment. J Educ Psychol. 2014;106(4):961–73. 10.1037/a0037116

[54] Wouters P, van Oostendorp H. A meta-analytic review of the role of instructional support in game-based learning. Comput Educ. 2013;60(1):412–25. 10.1016/j.compedu.2012.07.018

[55] Schwonke R. Metacognitive load – Useful, or extraneous concept? Metacognitive and self-regulatory demands in computer-based learning. J Educ Technol Soc. 2015;18(4):172–84.

[56] Seufert T. The interplay between self-regulation in learning and cognitive load. Educ Res Rev. 2018;24:116–29. 10.1016/j.edurev.2018.03.004

[57] Valcke M. Cognitive load: updating the theory? Learn Instr. 2002;12(1):147–54. 10.1016/S0959-4752(01)00022-6

[58] Perkins DN, Salomon G. Transfer of learning. Int Encyclopedia Educ. 1992;2:6452–7.

[59] Hajian S. Transfer of Learning and Teaching: A Review of Transfer Theories and Effective Instructional Practices. IAFOR J Educ. 2019;7:93–111. 10.22492/ije.7.1.06

[60] Basu S, Biswas G, Kinnebrew J. Learner modeling for adaptive scaffolding in a Computational Thinking-based science learning environment. User Model User-Adap Interact. 2017;26:5–3. 10.1007/s11257-017-9187-0

[61] Game WN, Theory E, Learning A. Game Engagement Theory and Adult Learning. Simul Gaming. 2011;42(5):596–609. 10.1177/1046878110378587

[62] Garris R, Ahlers R, Driskell JE. Games, motivation, and learning: a research and practice model. Simul Gaming. 2002;33(4):441–67. 10.1177/1046878102238607

[63] Barzilai S, Blau I. Scaffolding game-based learning: Impact on learning achievements, perceived learning, and game experiences. Comput Educ. 2014;70:65–79. 10.1016/j.compedu.2013.08.003

[64] Charsky D, Ressler W. “Games are made for fun”: Lessons on the effects of concept maps in the classroom use of computer games. Comput Educ. 2011;56(3):604–15. 10.1016/j.compedu.2010.10.001

[65] Broza O, Barzilai S, editors. When the mathematics of life meets school mathematics: Playing and learning on the “my money” website. Learning in the technological era: Proceedings of the sixth chais conference on instructional technologies research; 2011.

[66] van Merriënboer JJG, Clark RE, de Croock MBM. Blueprints for complex learning: The 4C/ID-model. Education Tech Research Dev. 2002;50(2):39–61. 10.1007/BF02504993

[67] Schoeber NHC, Linders M, Binkhorst M, De Boode W-P, Draaisma JMT, Morsink M, et al. Healthcare professionals’ knowledge of the systematic ABCDE approach: a cross-sectional study. BMC Emerg Med. 2022;22(1):202. 36510149 10.1186/s12873-022-00753-y PMC9743501

[68] Faber TJE, Dankbaar MEW, van Merriënboer JJG. Four-Component Instructional Design Applied to a Game for Emergency Medicine. In: Brooks AL, Brahman S, Kapralos B, Nakajima A, Tyerman J, Jain LC, editors. Recent Advances in Technologies for Inclusive Well-Being: Virtual Patients, Gamification and Simulation. Cham: Springer International Publishing; 2021. p. 65–82.

[69] Paas FG. Training strategies for attaining transfer of problem-solving skill in statistics: a cognitive-load approach. J Educ Psychol. 1992;84(4):429. 10.1037/0022-0663.84.4.429

[70] Rovers SFE, Clarebout G, Savelberg HHCM, de Bruin ABH, van Merriënboer JJG. Granularity matters: comparing different ways of measuring self-regulated learning. Metacogn Learn. 2019;14(1):1–19. 10.1007/s11409-019-09188-6

[71] Dankbaar MEW, Stegers-Jager KM, Baarveld F, Merrienboer JJGV, Norman GR, Rutten FL, et al. Assessing the assessment in emergency care training. PloS one. 2014;9(12):e114663-e. 25521702 10.1371/journal.pone.0114663 PMC4270684

[72] R Core Team. R: A language and environment for statistical computing. R Foundation for Statistical Computing; 2021. https://www.R-project.org/.

[73] RStudio Team. RStudio: Integrated Development Environment for R. Boston: RStudio, Inc.; 2018.

[74] Makowski D, Ben-Shachar MS, Patil I, Lüdecke D. Methods and algorithms for correlation analysis in R. J Open Source Softw. 2020;5(51):2306. 10.21105/joss.02306

[75] Magezi DA. Linear mixed-effects models for within-participant psychology experiments: an introductory tutorial and free, graphical user interface (LMMgui). Front Psychol. 2015;6:2. 25657634 10.3389/fpsyg.2015.00002 PMC4302710

[76] Bates D, Mächler M, Bolker B, Walker S. Fitting Linear Mixed-Effects Models Using lme4. J Stat Softw. 2015;1(1):2015.

[77] Wu L, Looi C-K. Agent prompts: Scaffolding for productive reflection in an intelligent learning environment. J Educ Technol Soc. 2012;15(1):339–53.

[78] Azevedo R, Cromley JG, Winters FI, Moos DC, Greene JA. Adaptive human scaffolding facilitates adolescents' self-regulated learning with hypermedia. Instr Sci. 2005;33:381–412. 10.1007/s11251-005-1273-8.

[79] Perry NE. Young children’s self-regulated learning and contexts that support it. J Educ Psychol. 1998;90:715–29. 10.1037/0022-0663.90.4.715

[80] Ericsson KA. Protocol Analysis and Expert Thought: Concurrent Verbalizations of Thinking during Experts' Performance on Representative Tasks. In K. A. Ericsson, N. Charness, P. J. Feltovich, & R. R. Hoffman (Eds.), The Cambridge handbook of expertise and expert performance (pp. 223–241). Cambridge University Press; 2006. 10.1017/CBO9780511816796.013.

[81] Cleary TJ. Emergence of Self-Regulated Learning Microanalysis. Handbook of Self-Regulation of Learning and Performance. 2017. p. 10513.

[82] Kok EM, Jarodzka H. Before your very eyes: the value and limitations of eye tracking in medical education. Med Educ. 2017;51(1):114–22. 27580633 10.1111/medu.13066

[83] Paas FGWC, van Merriënboer JJG, Adam JJ. Measurement of Cognitive Load in Instructional Research. Percept Mot Skills. 1994;79(1):419–30. 7808878 10.2466/pms.1994.79.1.419

[84] Haji FA, Rojas D, Childs R, de Ribaupierre S, Dubrowski A. Measuring cognitive load: performance, mental effort and simulation task complexity. Med Educ. 2015;49(8):815–27. 26152493 10.1111/medu.12773

[85] Klepsch M, Seufert T. Making an Effort Versus Experiencing Load. Front Educ. 2021;6:645284. 10.3389/feduc.2021.645284

[86] Krieglstein F, Beege M, Rey GD, Sanchez-Stockhammer C, Schneider S. Development and validation of a theory-based questionnaire to measure different types of cognitive load. Educ Psychol Rev. 2023;35(1):9. 10.1007/s10648-023-09738-0

[87] Klepsch M, Schmitz F, Seufert T. Development and Validation of Two Instruments Measuring Intrinsic, Extraneous, and Germane Cognitive Load. Front Psychol. 2017;8:1997. 29201011 10.3389/fpsyg.2017.01997 PMC5696680

[88] Sweller J. Element interactivity and intrinsic, extraneous, and germane cognitive load. Educ Psychol Rev. 2010;22:123–38. 10.1007/s10648-010-9128-5

