# Supplementary material for: Effects of adaptive scaffolding on performance, cognitive load and engagement in game-based learning: a randomized controlled trial
Source: BMC Med Educ. 2024 Aug 29;24:943. doi: 10.1186/s12909-024-05698-3 (PMC11360721; doi:10.1186/s12909-024-05698-3)
Supplement: Supplementary file 1 — Supplementary Material 1. [file 12909_2024_5698_MOESM1_ESM.docx]

## Demographics questionnaire

To be completed prior to participating in the study

1. Age: ____ year
2. Gender: male / female/ other
3. Year of study:
4. University:
5. How much hands-on, real-life experience do you have caring for acutely ill patients? Include cases where you only observed or assisted in your count.
   0 cases 1-2 cases 3-5 cases 6-10 cases >10 cases

## Assessment instrument for emergency care skill

| Participant code |  |
| --- | --- |
| Date | __ /__ / ____ |
| University |  |
| Assessor |  |
| Scenario  Note: 1 and 2 are used in the immediate assessment,  3 and 4 in the delayed assessment | 1 / 2 / 3 / 4 |

|  | **Competency Scale** | **very weak** | **weak** | **insufficient** | **questionable** | **sufficient** | **good** | **excellent** |
| --- | --- | --- | --- | --- | --- | --- | --- | --- |
|  | **ABCDE approach** |  |  |  |  |  |  |  |
| 1 | Uses ABCDE approach on initial assessment |  |  |  |  |  |  |  |
| 2 | Uses ABCDE approach on initial treatment |  |  |  |  |  |  |  |
| 3 | Uses re-assessment properly |  |  |  |  |  |  |  |
|  | **Additional actions** |  |  |  |  |  |  |  |
| 4 | Requests additional diagnostic studies |  |  |  |  |  |  |  |
| 5 | Proposes a working diagnoses |  |  |  |  |  |  |  |
| 6 | Consults specialist when needed |  |  |  |  |  |  |  |

**Global Performance Scale
Assessment of independent function in caring for acutely ill patients in the Emergency Department**:

*Worst* 1 2 3 4 5 6 7 8 9 10 *Best*

**Passed/Failed**

## Game engagement questionnaire

**5 point scale (1= fully disagree, 2= disagree, 3= neutral, 4= agree, 5= fully agree)**

Choose the assertion which fits best your opinion on the game you just played

|  |  | **Fully disagree** | **Disagree** | **Neutral** | **Agree** | **Fully agree** |
| --- | --- | --- | --- | --- | --- | --- |
|  |  |  |  |  |  |  |
| 1 | I felt actively involved with the patient scenarios |  |  |  |  |  |
| 2 | My attention was completely on the game scenarios |  |  |  |  |  |
| 3 | I liked playing the game |  |  |  |  |  |
| 4 | I felt the content of the game to be instructive |  |  |  |  |  |
| 5 | I was able to concentrate well during play |  |  |  |  |  |
| 6 | I enjoyed this way of learning |  |  |  |  |  |
| 7 | During gameplay, I felt like I was the doctor in charge in the ED |  |  |  |  |  |
| 8 | I could find out for myself what did and did not work well |  |  |  |  |  |
| 9 | I regularly felt stressed during playing the scenarios |  |  |  |  |  |

Do you have any comments to make on the game?

Strong points:

Points of improvement:
