# Supplementary material for: Effects of adaptive scaffolding on performance, cognitive load and engagement in game-based learning: a randomized controlled trial
Source: BMC Med Educ. 2024 Aug 29;24:943. doi: 10.1186/s12909-024-05698-3 (PMC11360721; doi:10.1186/s12909-024-05698-3)
Supplement: Supplementary file 2 — Supplementary Material 2. [file 12909_2024_5698_MOESM2_ESM.pdf]

82. Cleary TJ. Emergence of Self-Regulated Learning Microanalysis. Handbook of Self-Regulation of Learning and Performance. 2017(10513).

83. Kok EM, Jarodzka H. Before your very eyes: the value and limitations of eye tracking in medical education. Med Educ. 2017;51(1):114-22.

84. Paas FGWC, van Merriënboer JJG, Adam JJ. Measurement of Cognitive Load in Instructional Research. Perceptual and Motor Skills. 1994;79(1):419-30.

85. Haji FA, Rojas D, Childs R, de Ribaupierre S, Dubrowski A. Measuring cognitive load: performance, mental effort and simulation task complexity. Med Educ. 2015;49(8):815-27.

86. Klepsch M, Seufert T. Making an Effort Versus Experiencing Load. Frontiers in Education. 2021;6.

87. Krieglstein F, Beege M, Rey GD, Sanchez-Stockhammer C, Schneider S. Development and Validation of a Theory-Based Questionnaire to Measure Different Types of Cognitive Load. Educational Psychology Review. 2023;35(1):9.

88. Klepsch M, Schmitz F, Seufert T. Development and Validation of Two Instruments Measuring Intrinsic, Extraneous, and Germane Cognitive Load. Frontiers in Psychology. 2017;8.

89. Sweller J. Element interactivity and intrinsic, extraneous, and germane cognitive load. Educational psychology review. 2010;22:123-38.

Supplementary files

Supplementary Table A. Comparing models including adaptive condition as a fixed effect with the model without adaptive condition

	Outcome measure	X_2	*df*	*p*	*R2m*	*R2c*
**Cognitive load**						
	Cognitive load	1,71	1	.191	.07	.36
**Learning performance**						
	Accuracy of decision making	0,12	1	.727	.08	.14
	Time to scenario completion	3,61	1	.057	.03	.25
	Time to vital interventions	0,19	1	.666	.00	.11
	Systematicity	0,14	1	.706	.00	.06
**Self-regulation**						
	Self-monitoring	3,53	1	.060	.03	.40
	Help-seeking	2,01	1	.156	.05	.16

Supplementary Table B. Estimates for adaptive scaffolding condition and learning performance

	Accuracy of decision making				Time until scenario completion (seconds)				Time to vital interventions (z-scores)				Systematicity in approach (%)			
Predictors	*Estimates*	*SE*	*CI*	*p*	*Estimates*	*SE*	*CI*	*p*	*Estimates*	*SE*	*CI*	*p*	*Estimates*	*SE*	*CI*	*p*
(Intercept)	500.98	10.65	480.05 – 521.92	**<0.001 ***	638.36	22.88	593.36 – 683.35	**<0.001 ***	0.00	0.15	-0.30 – 0.30	0.989	0.80	0.01	0.78 – 0.81	**<0.001 ** **
Number of scenarios played previously	16.10	2.79	10.61 – 21.58	**<0.001 ***	-8.79	4.83	-18.28 – 0.69	0.069	-0.00	0.04	-0.08 – 0.08	0.923	-0.00	0.00	-0.00 – 0.00	0.946
Support condition	3.95	11.46	-18.59 – 26.49	0.730	52.60	27.71	-1.89 – 107.09	0.058	0.06	0.14	-0.22 – 0.35	0.661	-0.00	0.01	-0.02 – 0.01	0.711
**Random Effects**																
σ^2	8619.10				25783.66				0.99				0.01			
τ_{00}	627.76 Participant identifier				7776.58 Participant identifier				0.00 Participant identifier				0.00 Participant identifier			
	0.00 pair				0.00 pair				0.12 pair				0.00 pair			
ICC																
N	32 pair				32 pair				30 pair				32 pair			
	63 Participant identifier				63 Participant identifier				49 Participant identifier				63 Participant identifier			
Observations	377				377				221				377			
Marginal R^2 / Conditional R^2	0.082 / NA				0.035 / NA				0.001 / NA				0.001 / NA			

Supplementary Table C. Estimates for adaptive scaffolding condition and SRL and cognitive load

	Cognitive load				Self-monitoring				Help-seeking			
Predictors	*Estimates*	*SE*	*CI*	*p*	*Estimates*	*SE*	*CI*	*p*	*Estimates*	*SE*	*CI*	*p*
(Intercept)	5.06	0.24	4.59 – 5.53	**<0.001**	1.56	0.27	1.03 – 2.09	**<0.001**	1.86	0.21	1.46 – 2.27	**<0.001**
Number of scenarios played previously	0.21	0.04	0.12 – 0.30	**<0.001**	0.05	0.05	-0.05 – 0.14	0.323	0.23	0.05	0.13 – 0.34	**<0.001**
Adaptive support condition	-0.36	0.29	-0.92 – 0.20	0.211	0.65	0.35	-0.03 – 1.34	0.062	-0.33	0.23	-0.78 – 0.13	0.160
Random Effects												
σ^2	1.45				2.42				2.93			
τ_{00}	0.65 Participant identifier				1.50 Participant identifier				0.35 Participant identifier			
	0.00 pair				0.00 pair				0.00 pair			
N	30 pair				32 pair				32 pair			
	49 Participant identifier				63 Participant identifier				63 Participant identifier			
Observations	244				377				377			
Marginal R^2 / Conditional R^2	0.101 / NA				0.044 / NA				0.061 / NA			

Supplementary Table D. Comparing models including tailored scaffolding as a fixed effect with the model without tailored scaffolding

	Outcome measure	X2	df	p	R2m	R2c
**Cognitive load**						
	Cognitive load	14,85	6	**.021**	.16	.45
**Learning performance**						
	Accuracy of decision making	14,36	6	**.026**	.12	.24
	Time to scenario completion	23,30	6	**.001**	.13	.36
	Time to vital interventions	24,51	6	**<.001**	.20	.26
	Systematicity	5,44	6	.489	.03	.16
**Self-regulation**						
	Self-monitoring	55,99	6	**<.001**	.15	.63
	Help-seeking	16,45	6	**.012**	.10	.28

Supplementary Table E. Effects of tailored scaffolding on learning performance in sessions played by the nonadaptive group

	Accuracy of decision making				Time until scenario completion (seconds)				Time to vital interventions (z-scores)				Systematicity in approach (%)			
Predictors	*Estimates*	*SE*	*CI*	*p*	*Estimates*	*SE*	*CI*	*p*	*Estimates*	*SE*	*CI*	*p*	*Estimates*	*SE*	*CI*	*p*
(Intercept)	551.55	27.73	496.83 – 606.26	**<0.001 ***	781.76	47.69	687.66 – 875.85	**<0.001 ***	0.46	0.42	-0.37 – 1.29	0.276	79.20	2.16	74.94 – 83.47	**<0.001 ***
Number of scenarios played previously	12.50	4.08	4.45 – 20.54	**0.003 ***	-23.42	6.72	-36.68 – -10.15	**0.001 ***	0.01	0.07	-0.12 – 0.14	0.917	0.02	0.32	-0.61 – 0.65	0.955
Tailored supportive information	-32.61	21.08	-74.20 – 8.97	0.124	-90.57	35.25	-160.13 – -21.02	**0.011 ***	-0.82	0.32	-1.45 – -0.19	**0.011 ***	0.27	1.65	-2.98 – 3.52	0.870
Tailored procedural information	-17.41	16.28	-49.53 – 14.72	0.287	-36.76	27.10	-90.23 – 16.72	0.177	0.32	0.25	-0.18 – 0.83	0.211	0.14	1.27	-2.37 – 2.66	0.910
**Random Effects**																
**σ2**	8481.39				23004.24				1.23				52.05			
**τ00**	1110.99 Participant identifier				7622.01 Participant identifier				0.08 Participant identifier				5.82 Participant identifier			
**ICC**	0.12				0.25				0.06				0.10			
**N**	32 Participant identifier				32 Participant identifier				25 Participant identifier				32 Participant identifier			
**Observations**	191				191				111				191			
Marginal R^2 / Conditional R^2	0.070 / 0.178				0.071 / 0.302				0.076 / 0.134				0.000 / 0.101			

Supplementary Table F. Effects of tailored scaffolding on cognitive load and SRL in sessions played by the nonadaptive group

	**Cognitive load**				**Self-monitoring**				**Help-seeking**			
Predictors	Estimates	SE	CI	*p*	*Estimates*	*SE*	*CI*	*p*	*Estimates*	*SE*	*CI*	*p*
(Intercept)	6.29	0.52	5.26 – 7.32	**<0.001**	2.32	0.44	1.46 – 3.17	**<0.001**	2.79	0.53	1.74 – 3.83	**<0.001**
Number of scenarios played previously	0.18	0.07	0.05 – 0.32	**0.009**	0.01	0.06	-0.10 – 0.12	0.892	0.15	0.08	0.00 – 0.30	**0.046**
Tailored supportive information	-0.88	0.34	-1.56 – -0.20	**0.012**	-0.85	0.30	-1.44 – -0.26	**0.005**	-0.13	0.40	-0.91 – 0.65	0.741
Tailored procedural information	-0.51	0.30	-1.10 – 0.09	0.093	0.12	0.23	-0.33 – 0.57	0.611	-0.81	0.31	-1.41 – -0.21	**0.009**
Random Effects												
σ2	1.30				1.61				2.95			
τ00	0.61 Participant identifier				1.43 Participant identifier				0.66 Participant identifier			
ICC	0.32				0.47				0.18			
N	23 Participant identifier				32 Participant identifier				32 Participant identifier			
Observations	110				191				191			
Marginal R^2 / Conditional R^2	0.128 / 0.405				0.026 / 0.484				0.065 / 0.235			

Supplementary Table G. Descriptive statistics by tailored supportive information

	Untailored supportive information (N=23)	Tailored supportive information (N=168)
**Age**		
Mean (SD)	23.48 (1.75)	22.90 (1.61)
Range	20.00 - 27.00	20.00 - 27.00
**Gender**		
Male	5 (21.7%)	38 (22.6%)
Female	17 (73.9%)	125 (74.4%)
Other	1 (4.3%)	5 (3.0%)
**Experience in emergency care**		
0 casus	8 (34.8%)	74 (44.0%)
1-2 cases	10 (43.5%)	51 (30.4%)
More than 2 cases	5 (21.7%)	43 (25.6%)
**Study year**		
Mean (SD)	4.61 (0.99)	4.28 (1.02)
Range	3.00 - 6.00	3.00 - 6.00
**Score on knowledge test (%)**		
Mean (SD)	83.98 (7.76)	85.68 (7.85)
Range	69.00 - 96.60	69.00 - 96.60
**Tailored procedural information (%)**		
Mean (SD)	78% (42%)	74% (44%)
**Accuracy**		
Mean (SD)	567.17 (79.40)	537.70 (102.96)
Range	429.00 - 688.00	260.00 - 800.00
**Systematicity in approach**		
Mean (SD)	78.97 (7.30)	79.69 (7.59)
Range	61.82 - 88.73	38.77 - 93.19

**Speed (absolute, time to scenario completion)**		
Mean (SD)	691.71 (181.91)	605.33 (177.16)
Range	384.47 - 900.00	152.20 - 900.00
**Speed (relative, time to complete critical interventions)**		
N-Miss	8	72
Mean (SD)	0.72 (2.20)	-0.11 (0.89)
Range	-0.37 - 6.96	-0.72 - 7.29
**Cognitive load**		
N-Miss	9	72
Mean (SD)	6.21 (0.89)	5.54 (1.51)
Range	5.00 - 7.00	2.00 - 9.00
**Help-seeking**		
Mean (SD)	2.39 (2.19)	2.45 (1.92)
Range	0.00 - 9.00	0.00 - 8.00
**Self-monitoring**		
Mean (SD)	2.26 (2.30)	1.60 (1.64)
Range	0.00 - 8.00	0.00 - 10.00

Note. *p* values are not provided because of the multi-level nature of the data. See text and supplementary tables E and F for estimates and standard errors.

Supplementary Table H. Descriptive statistics by tailored procedural information

	Untailored procedural information (N=49)	Tailored supportive information (N=142)
**Age**		
Mean (SD)	22.73 (1.73)	23.05 (1.60)
Range	20.00 - 27.00	20.00 - 27.00
**Gender**		
Male	11 (22.4%)	32 (22.5%)
Female	36 (73.5%)	106 (74.6%)
Other	2 (4.1%)	4 (2.8%)
**Experience in emergency care**		
0 casus	23 (46.9%)	59 (41.5%)
1-2 cases	15 (30.6%)	46 (32.4%)
More than 2 cases	11 (22.4%)	37 (26.1%)
**Study year**		
Mean (SD)	4.20 (1.08)	4.36 (1.00)
Range	3.00 - 6.00	3.00 - 6.00
**Score on knowledge test (%)**		
Mean (SD)	84.05 (7.90)	85.96 (7.79)
Range	69.00 - 96.60	69.00 - 96.60
**Tailored supportive information (%)**		
Mean (SD)	0.90 (0.31)	0.87 (0.33)
**Accuracy**		
Mean (SD)	565.29 (112.24)	532.95 (95.42)
Range	345.00 - 800.00	260.00 - 746.00
**Systematicity in approach**		
Mean (SD)	79.47 (8.84)	79.65 (7.07)
Range	38.77 - 90.14	58.67 - 93.19
